# Supplementary material for: A partially disordered crystallographic shear block structure as fast-charging negative electrode material for lithium-ion batteries
Source: Nat Commun. 2025 Jul 15;16:6507. doi: 10.1038/s41467-025-61646-9 (PMC12264014; doi:10.1038/s41467-025-61646-9)
Supplement: Supplementary file 1 — Supplementary Information [file 41467_2025_61646_MOESM1_ESM.pdf]

--- Supplementary Information ---

A Partially Disordered Crystallographic Shear Block Structure as Fast-Charging Negative Electrode Material for Lithium-Ion Batteries

Yanchen Liu[1], Ana Guilherme Buzanich[2], Luciano A. Montoro[3], Hao Liu[4], Ye Liu[1], Franziska Emmerling[2], Patrícia A. Russo[1]*, Nicola Pinna[1]*

1 Department of Chemistry and The Center for the Science of Materials Berlin, Humboldt-Universität zu Berlin, Brook-Taylor-Str. 2, 12489 Berlin, Germany

2 Bundesanstalt für Materialforschung und -prüfung (BAM), Richard-Willstätter-Straße 11, 12489, Berlin, Germany

3 Universidade Federal de Minas Gerais, Department of Chemistry, Belo Horizonte, MG 31270-901, Brazil

4 Institute for Applied Materials (IAM), Karlsruhe Institute of Technology (KIT), Hermann-von-Helmholtz-Platz 1, 76344 Eggenstein-Leopoldshafen, Germany

*Corresponding authors: patricia.russo@hu-berlin.de, nicola.pinna@hu-berlin.de

Supplementary Methods

Electrochemical measurements:

GITT was carried out on a Land CT2001A battery test system in a voltage range of 1.0-3.0 V, which was tested by repeatedly applying current pulses at 0.1 C for 20 min and 120 min for relaxation. 10 data points were recorded per second. Based on Fick's first law[1], the electric current (I) can be expressed as:

$$I = \left(-D\frac{\partial c_{\mathrm{i}}}{\partial x}\bigg|_{x=0}\right) SZ_{\mathrm{i}}q \tag{1}$$

where I is the constant current pulse (I_0), D is the diffusion coefficient of species i (Li^+), x represents the distance coordinate, S is the area of the electrode, z_i is the charge number ($z_i = 1$ for Li), and q is the elementary charge ($q = 1.602\times10^{-19}$ C). Equation (1) shows that the current is equal to the number of charge carriers transported at the phase boundary of the electrode and the electrolyte ($x = 0$).

The concentration gradient, $\partial c_{\mathrm{Li}}/\ \partial x$, is a function of x and time (t). The diffusion coefficient is calculated with Fick's second law:

$$\frac{\partial c_{\mathrm{i}}(x,t)}{\partial_t} = D\frac{\partial^2 c_{\mathrm{i}}(x,t)}{\partial x^2} \tag{2}$$

with the initial and boundary conditions:

$$c_{\mathrm{i}}(x, t=0) = c_0\ (0 \leq\ x \leq L) \tag{3}$$

$$-D\frac{\partial c_{\mathrm{i}}}{\partial x}\bigg|_{x=0} = \frac{I_0}{Sz_{\mathrm{i}}q} \tag{4}$$

$$\frac{\partial c_{\mathrm{i}}}{\partial x}\bigg|_{x=L} = 0\ (t\ \geq 0) \tag{5}$$

Equation (3) is the initial condition at equilibrium. Equations (4) and (5) are boundary conditions at $x = 0$ and $x = L$, respectively. The solution of the differential Equation (2) under conditions (3-5) can be determined as follows for x = 0:

$$c_{\mathrm{i}}(x=0,t) = c_0\ + \frac{2I_0\sqrt{t}}{Sz_{\mathrm{i}}q\sqrt{D}}\sum_{n=0}^{\infty}\left(ierfc\left[\frac{nL}{\sqrt{Dt}}\right] + ierfc\left[\frac{(n+1)L}{\sqrt{Dt}}\right]\right) \tag{6}$$

where $ierfc$(z) = $[\pi^{-0.5}\exp(-z^2)] - z[1 - erf(z)]$, and $erf(z)$ is the error function. Considering that t $\ll L^2/D$, Equation (6) can be expressed as:

$$\frac{dc_{\mathrm{i}}(x=0,t)}{d\sqrt{t}} = \frac{2I_0\sqrt{t}}{Sz_{\mathrm{i}}q\sqrt{D}}\left(\mathrm{t} \ll \frac{L^2}{D}\right) \tag{7}$$

If the volume change with composition is neglected, and the relationship between the concentration and compositions is:

$$V_M dc_{\mathrm{i}} = N_A d\delta \tag{8}$$

where V_M is the molar volume of the electrode material ($\mathrm{cm^3\ mol^{-1}}$) and N_A is Avogadro's number ($6.02\times10^{23}\ \mathrm{mol^{-1}}$), insertion of equation (8) into equation (7) and expanding by dE, results in the following equation (9):

$$\frac{dE}{d\sqrt{t}} = \frac{2V_M I_0}{SFz_{\mathrm{i}}\sqrt{D\pi}}\frac{dE}{d\sigma}\left(\mathrm{t} \ll \frac{L^2}{D}\right) \tag{9}$$

The diffusion coefficient can then be obtained from Equation (9) as follows:

$$D = \frac{4}{\pi}\left(\frac{V_M I_0}{SFz_{\mathrm{i}}}\right)^2\left[\frac{dE}{d\delta}\Big/\frac{dE}{d\sqrt{t}}\right]^2\left(\mathrm{t} \ll \frac{L^2}{D}\right) \tag{10}$$

where F is Faraday's constant ($96485\ \mathrm{C\ mol^{-1}}$). dE / $d\delta$ and dE / $d\sqrt{t}$ represent the change in the steady-state voltage after subtracting the IR drop and transient change in voltage, respectively. When using a sufficiently small current, dE / $d\delta$ could be replaced by the ratio of the finite quantities, ΔE_s / $\Delta\delta$. Moreover, if E vs. $\sqrt{t}$ exhibits the linear behavior, it can be expressed as ΔE_τ / $\sqrt{\tau}$. The stoichiometry changes during the equilibration process in the titration step of Li^+ can be determined as follows:

$$\Delta\delta = \frac{\tau M_B I_0}{Fz_{\mathrm{i}} m_B} \tag{11}$$

where m_B and M_B are the mass of active material and its molecular weight, respectively. Then, equation (10) can be transformed into the following equation (12):

$$D = \frac{4}{\pi\tau}\left(\frac{m_B V_M}{M_B S}\right)^2\left(\frac{\Delta E_s}{\Delta E_\tau}\right)^2\left(\tau \ll \frac{L^2}{D}\right) \tag{12}$$

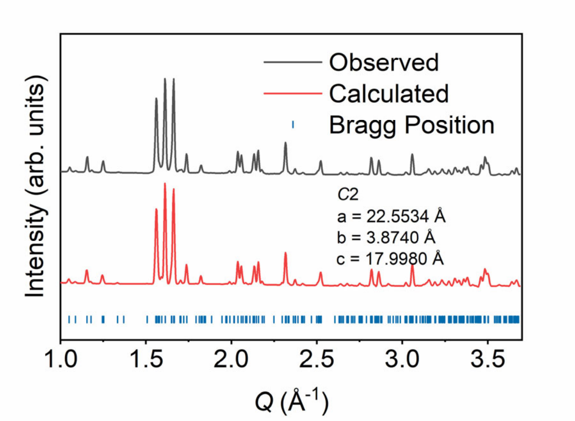

Supplementary Figure 1. Rietveld refinement of the XRD pattern of *m*-$Nb_{12}WO_{33}$ (R_{wp} = 9.6%).

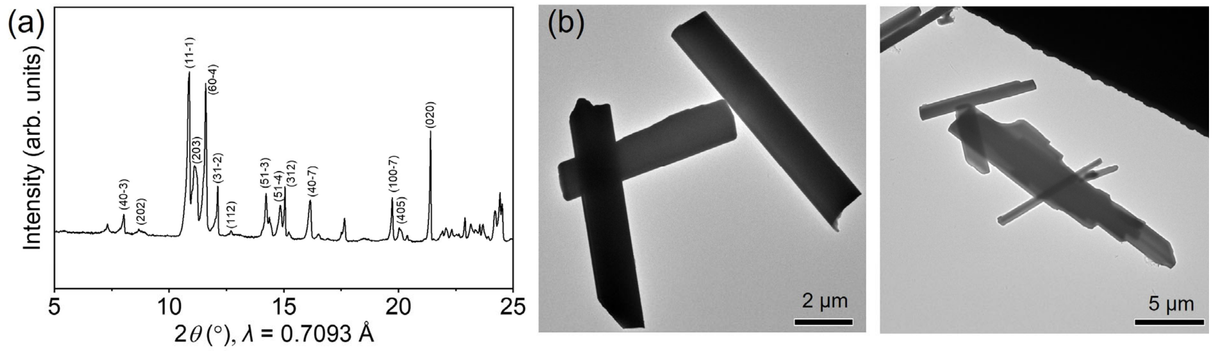

Supplementary Figure 2. (a) XRD pattern and (b) TEM images of the larger *m*-$Nb_{12}WO_{33}$ crystals.

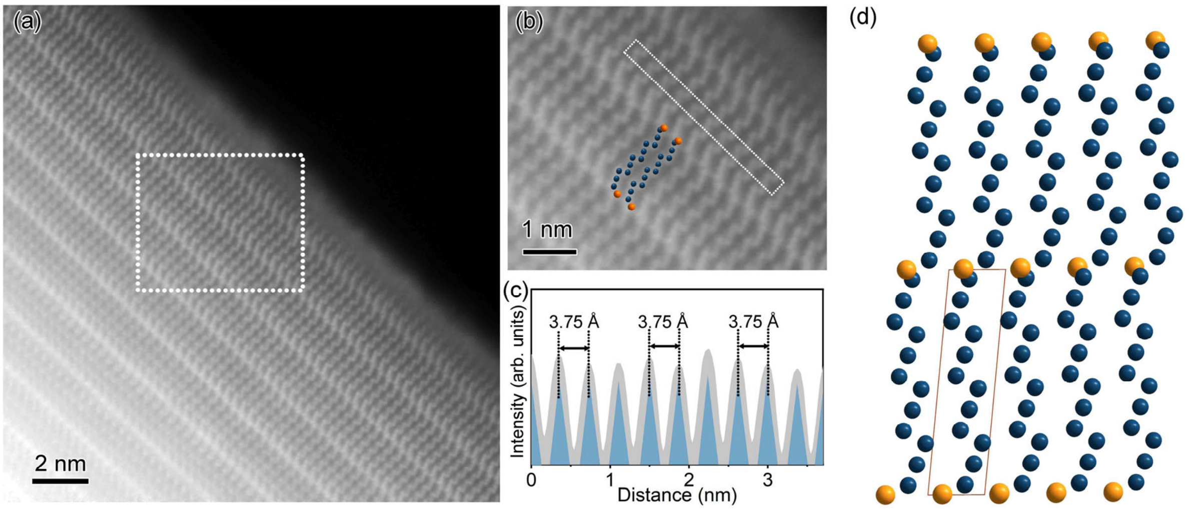

Supplementary Figure 3. (a) AC-STEM image of *m*-$Nb_{12}WO_{33}$ along the [1-10] zone axis. (b) Magnified image of the region delimited in (a). (c) Line profile of the region delimited in (b). (d) Schematic representation of the *m*-$Nb_{12}WO_{33}$ structure viewed along the a-axis (blue and orange spheres represent the Nb and W atoms, respectively).

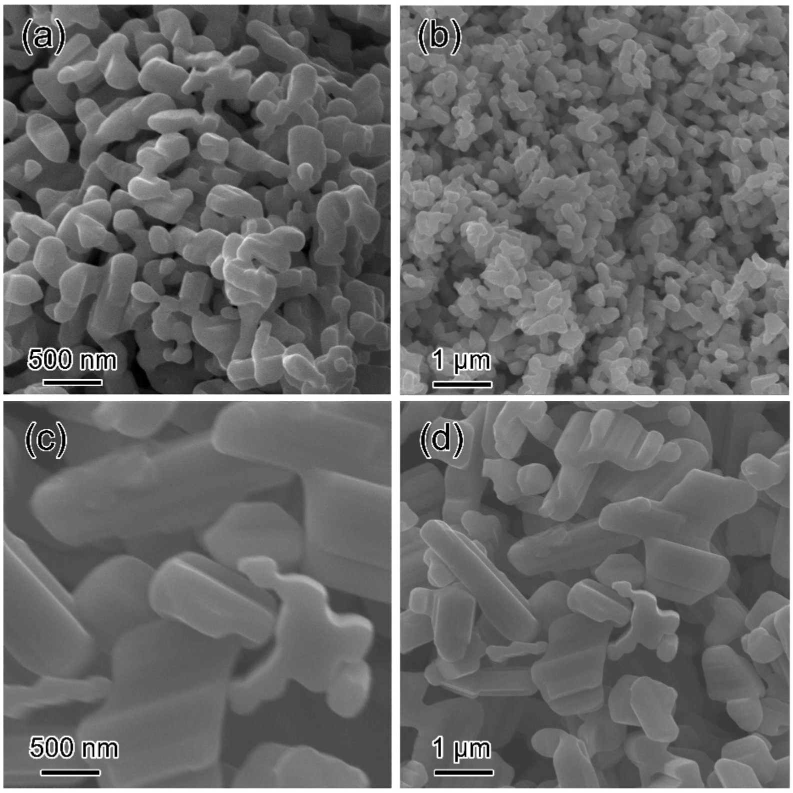

Supplementary Figure 4. (a) and (b) SEM images of *dt*-$Nb_{12}WO_{33}$. (c) and (d) SEM images of *m*-$Nb_{12}WO_{33}$.

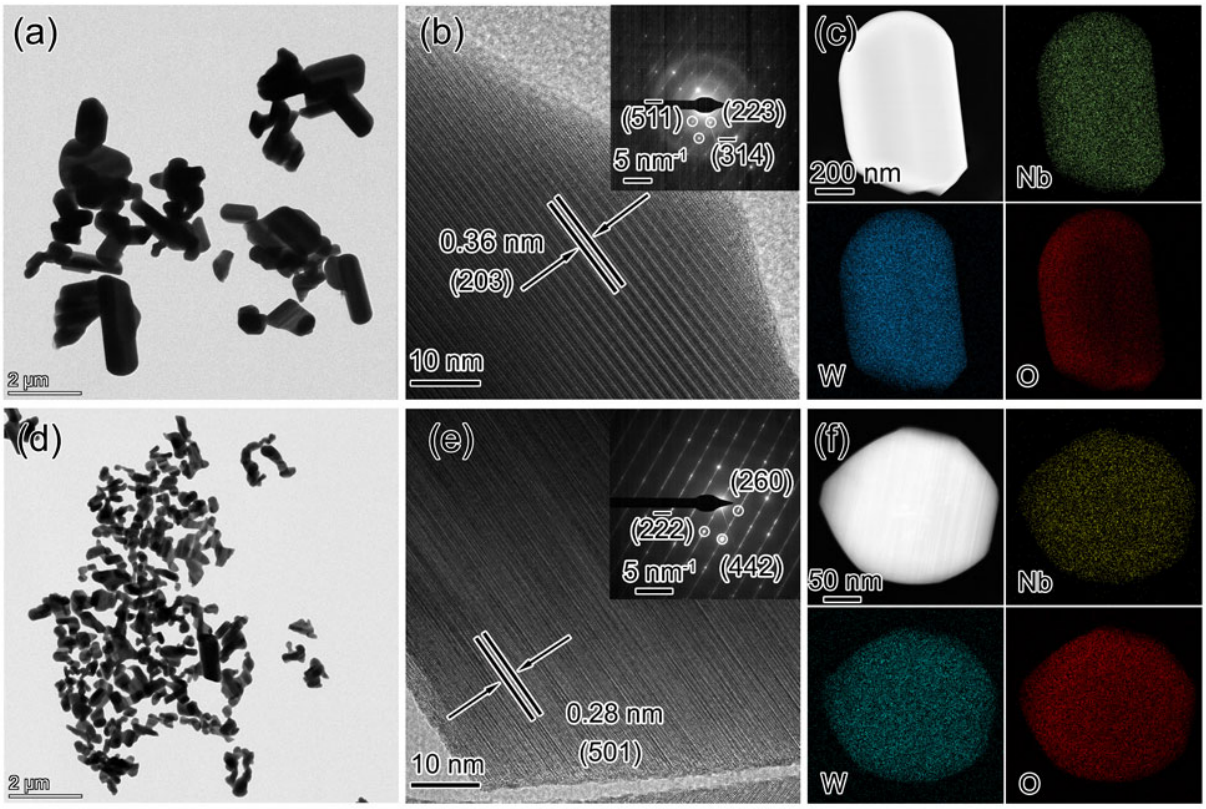

Supplementary Figure 5. (a) TEM, (b) HR-TEM, SAED and (c) EDS mapping images of *m*-$Nb_{12}WO_{33}$. (d) TEM, (e) HRTEM, SAED and (f) EDS mapping images of *dt*-$Nb_{12}WO_{33}$.

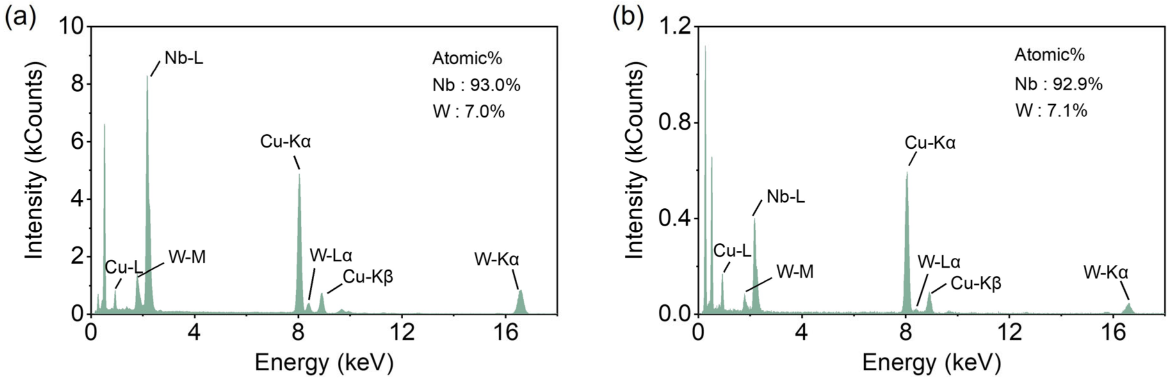

Supplementary Figure 6. EDS spectra of (a) *m*-$Nb_{12}WO_{33}$ and (b) *dt*-$Nb_{12}WO_{33}$.

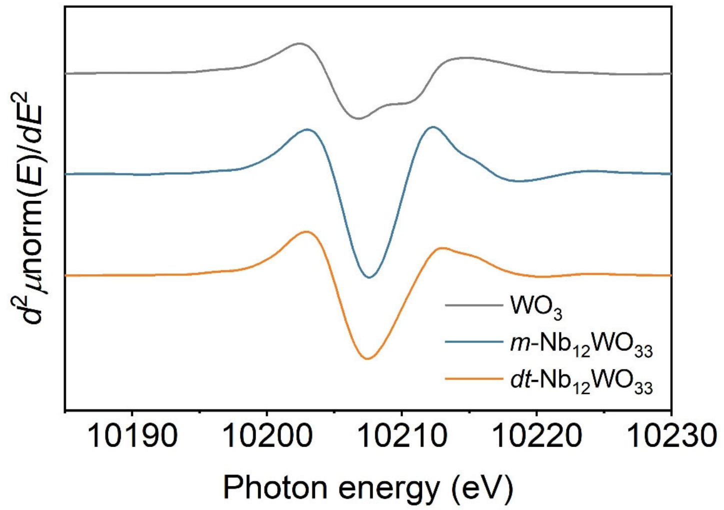

Supplementary Figure 7. Second derivatives of the W L_{III}-edge XANES spectra of WO_3, *m*-$Nb_{12}WO_{33}$ and *dt*-$Nb_{12}WO_{33}$.

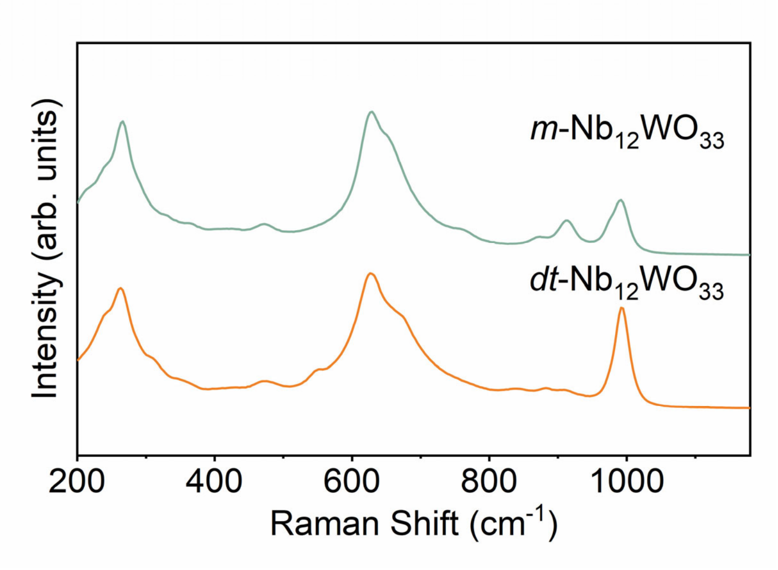

Supplementary Figure 8. Raman spectra of *m*-$Nb_{12}WO_{33}$ and *dt*-$Nb_{12}WO_{33}$.

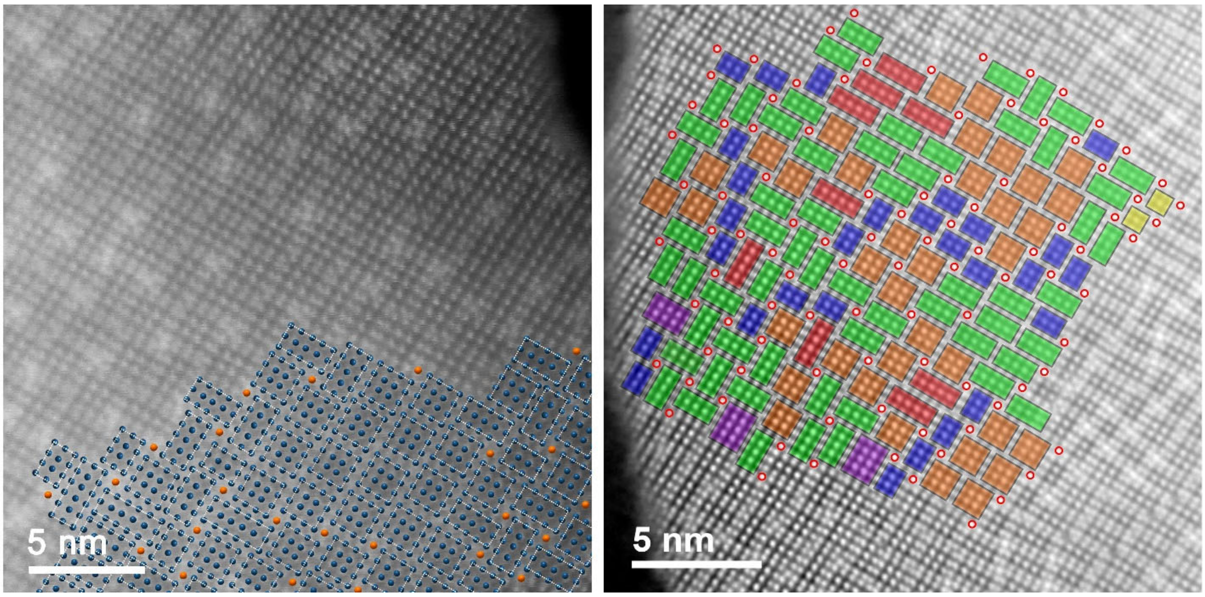

Supplementary Figure 9. AC-STEM images of *dt*-$Nb_{12}WO_{33}$ viewed along the [001] zone axis, showing blocks of different sizes (orange, blue, green, red, purple, yellow squares represent 4x4, 4x3, 5x3, 6x3, 5x4, and 3x3 blocks, respectively.)

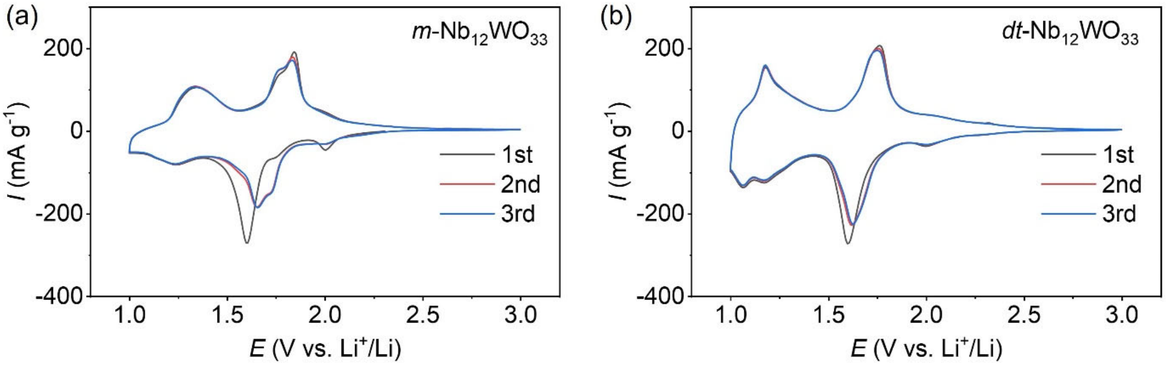

Supplementary Figure 10. CV curves of (a) *m*-$Nb_{12}WO_{33}$ and (b) *dt*-$Nb_{12}WO_{33}$ electrodes at a scanning rate of 0.1 mV s^{-1} within 1.0 – 3.0 V.

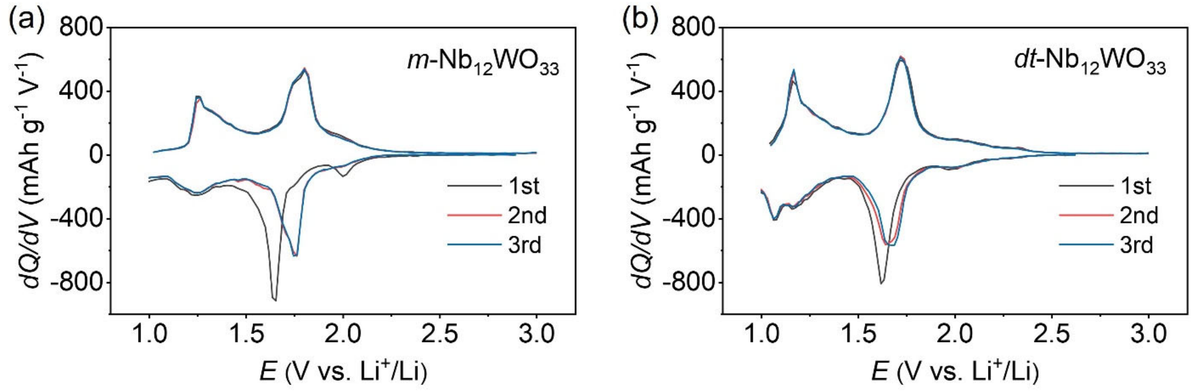

Supplementary Figure 11. dQ/dV curves of the initial three cycles of (a) *m*-$Nb_{12}WO_{33}$ and (b) *dt*-$Nb_{12}WO_{33}$ electrodes measured within a voltage window of 1.0–3.0 V at 0.5 C.

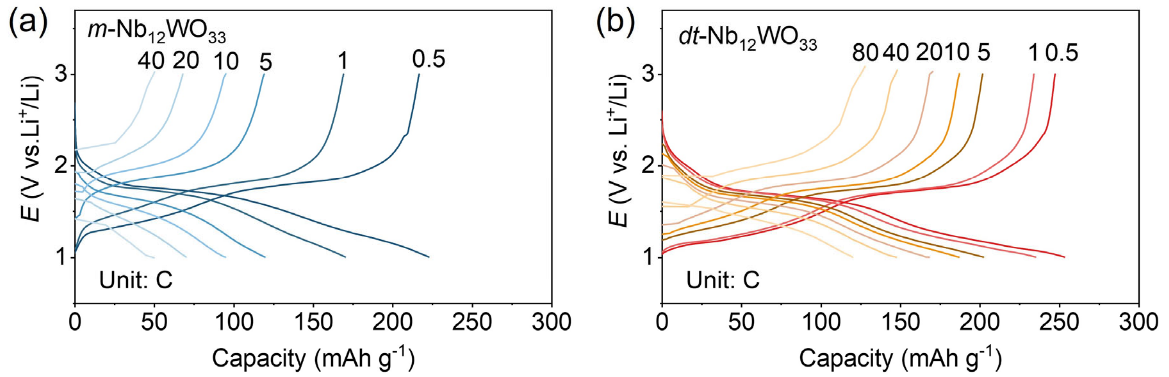

Supplementary Figure 12. Discharge-charge curves of (a) *m*-$Nb_{12}WO_{33}$ and (b) *dt*-$Nb_{12}WO_{33}$ at various rates.

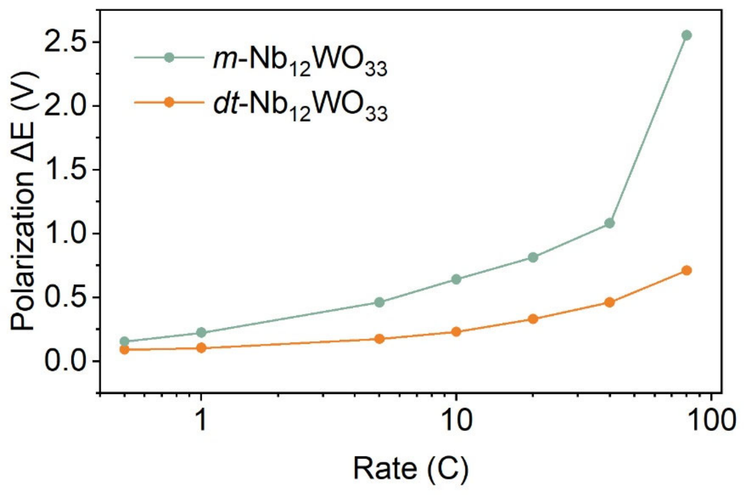

Supplementary Figure 13. Average voltage polarization of *m*-$Nb_{12}WO_{33}$ and *dt*-$Nb_{12}WO_{33}$ at various rates.

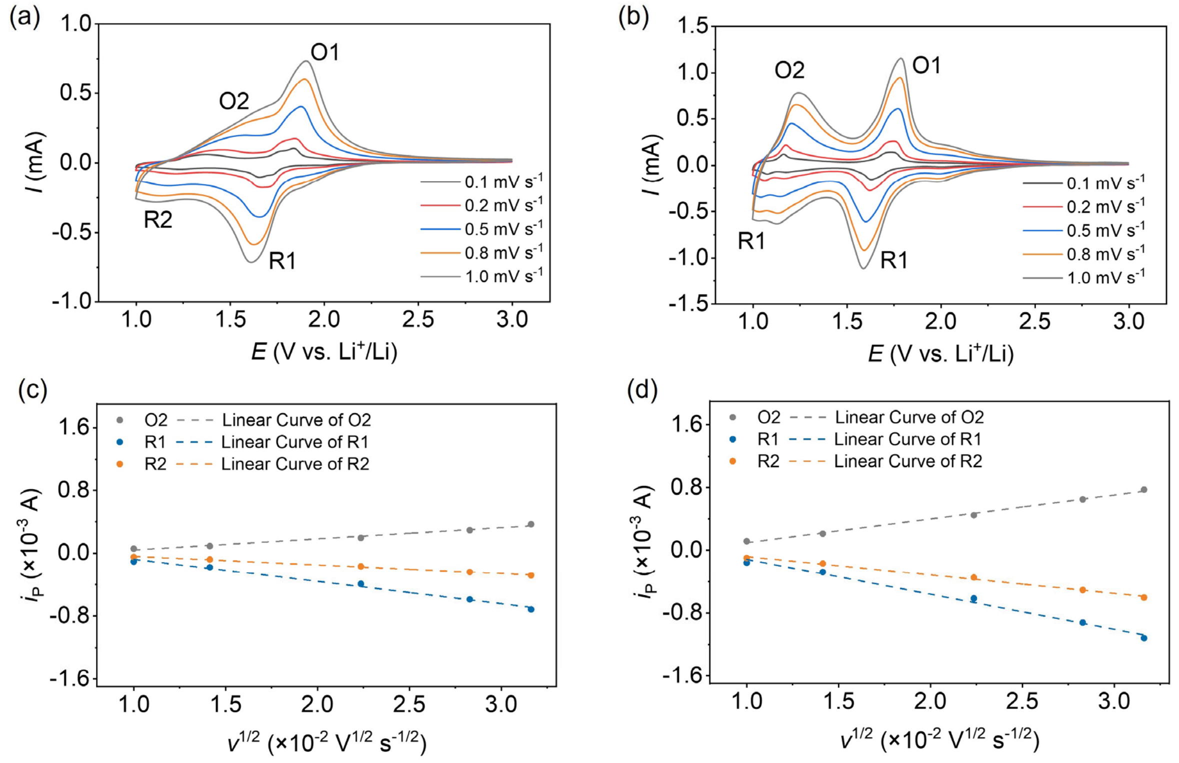

Supplementary Figure 14. CV curves at different scan rates for (a) *m*-$Nb_{12}WO_{33}$ and (b) *dt*-$Nb_{12}WO_{33}$ within 1.0 – 3.0 V. Linear relationship between the peak current (i_P) and the square root of the scan rate ($v^{1/2}$) for peak O2, R1 and R2 for (c) *m*-$Nb_{12}WO_{33}$ and (d) *dt*-$Nb_{12}WO_{33}$.

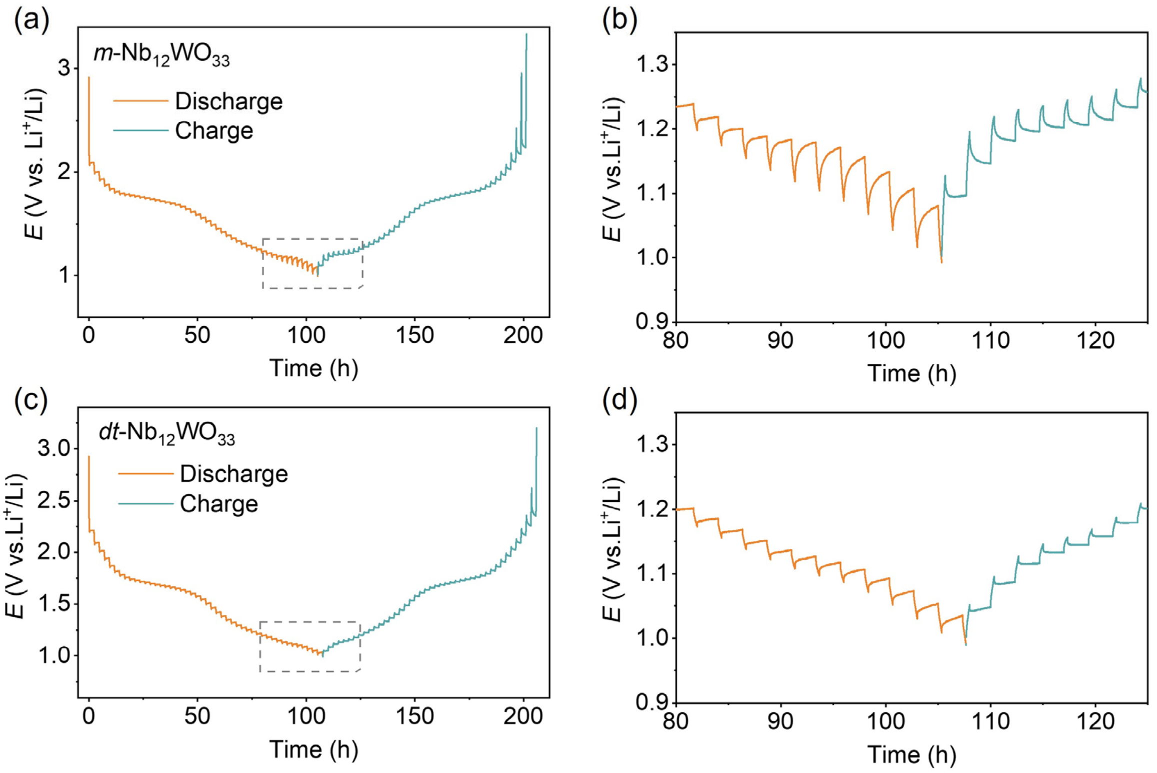

Supplementary Figure 15. Discharge-charge GITT profiles of (a) *m*-$Nb_{12}WO_{33}$ and (b) region delimited by the grey line in (a). Discharge-charge GITT profiles of (c) *dt*-$Nb_{12}WO_{33}$ and (d) region delimited by the grey line in (c).

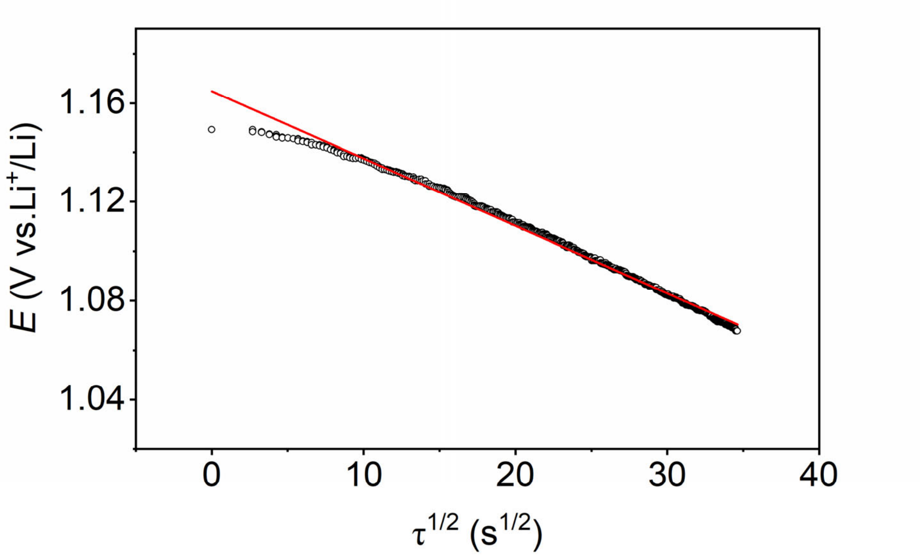

Supplementary Figure 16. Linear fit of the voltage (vs. Li^+/Li) *vs*. the square root of $\tau^{1/2}$ in GITT for the *m*-$Nb_{12}WO_{33}$ electrode at 1.15 V during the discharge process.

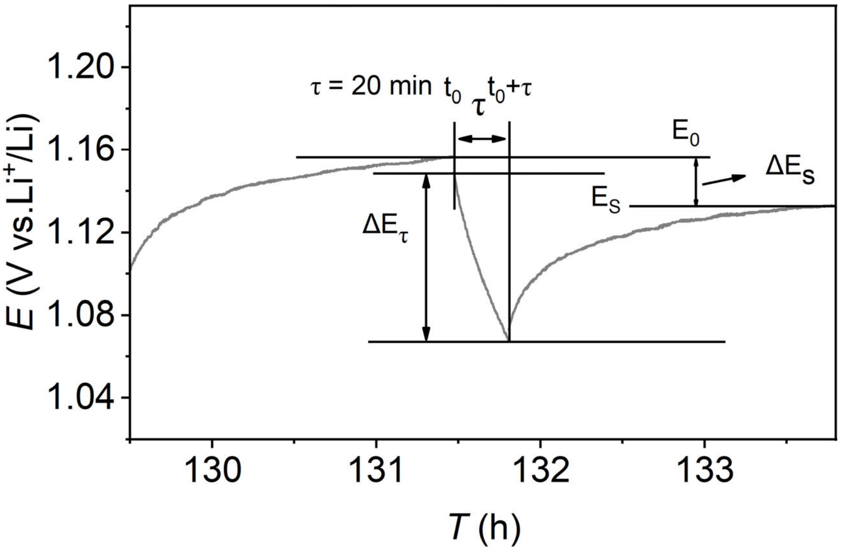

Supplementary Figure 17. Current step diagram at 1.15 V of *m*-$Nb_{12}WO_{33}$ during the discharge process.

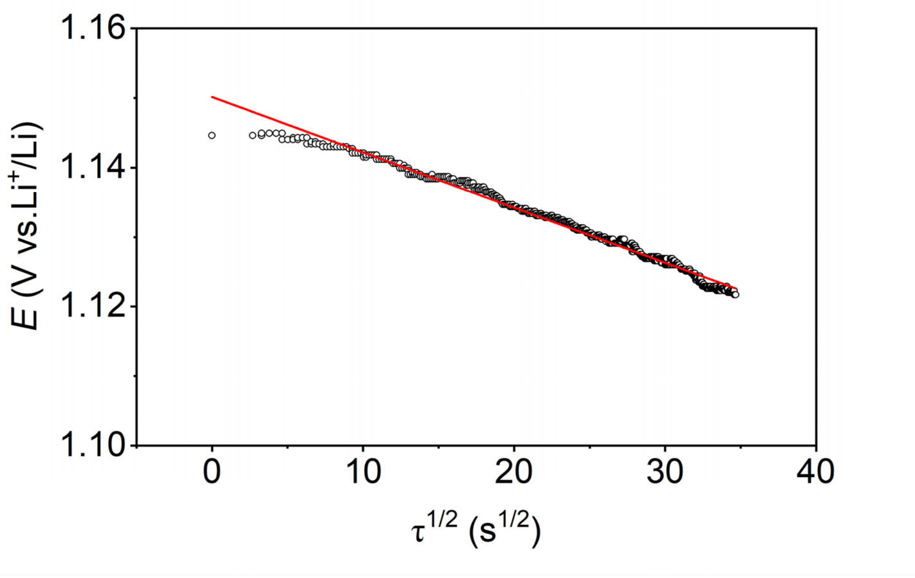

Supplementary Figure 18. Linear fit of the voltage (vs. Li^+/Li) *vs*. the square root of $\tau^{1/2}$ in GITT for *dt*- $Nb_{12}WO_{33}$ electrode at 1.15 V during the discharge process. The voltage shows a linear relationship with the square root of $\tau^{1/2}$, suggesting a diffusion-dominated process in *dt*-$Nb_{12}WO_{33}$.

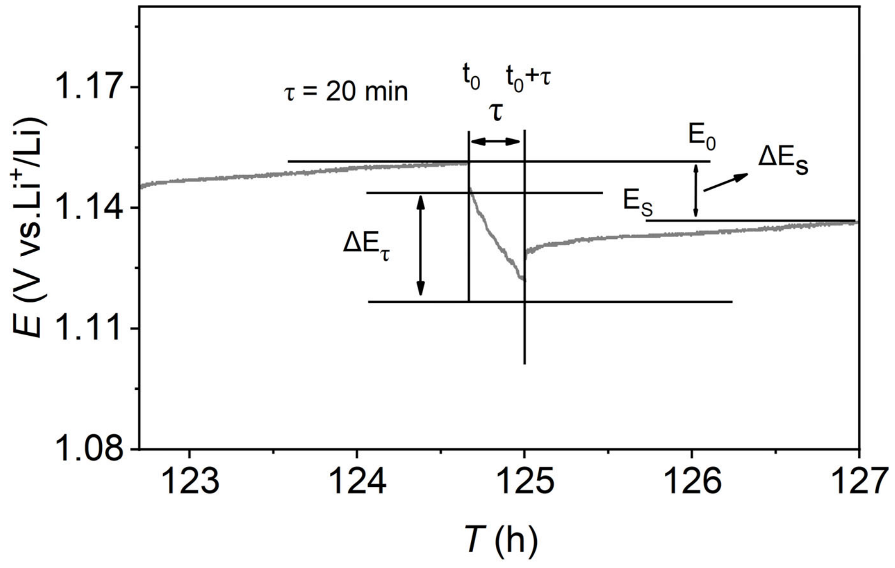

Supplementary Figure 19. Current step diagram at 1.15 V of *dt*-$Nb_{12}WO_{33}$ during the discharge process.

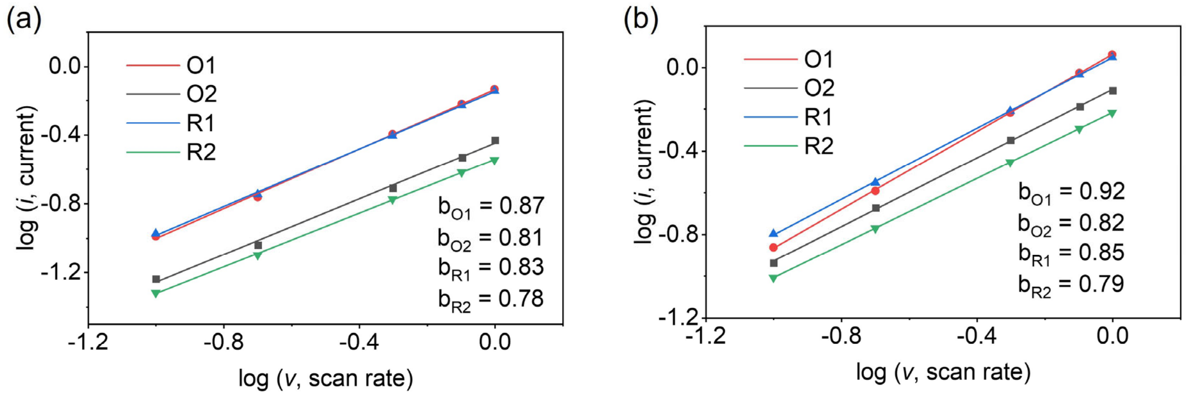

Supplementary Figure 20. Linear relationship between log(i) and log(v) for peaks O1, O2, R1 and R2 for (a) *m*-$Nb_{12}WO_{33}$ and (b) *dt*-$Nb_{12}WO_{33}$ obtained from CV curves at different scan rates.

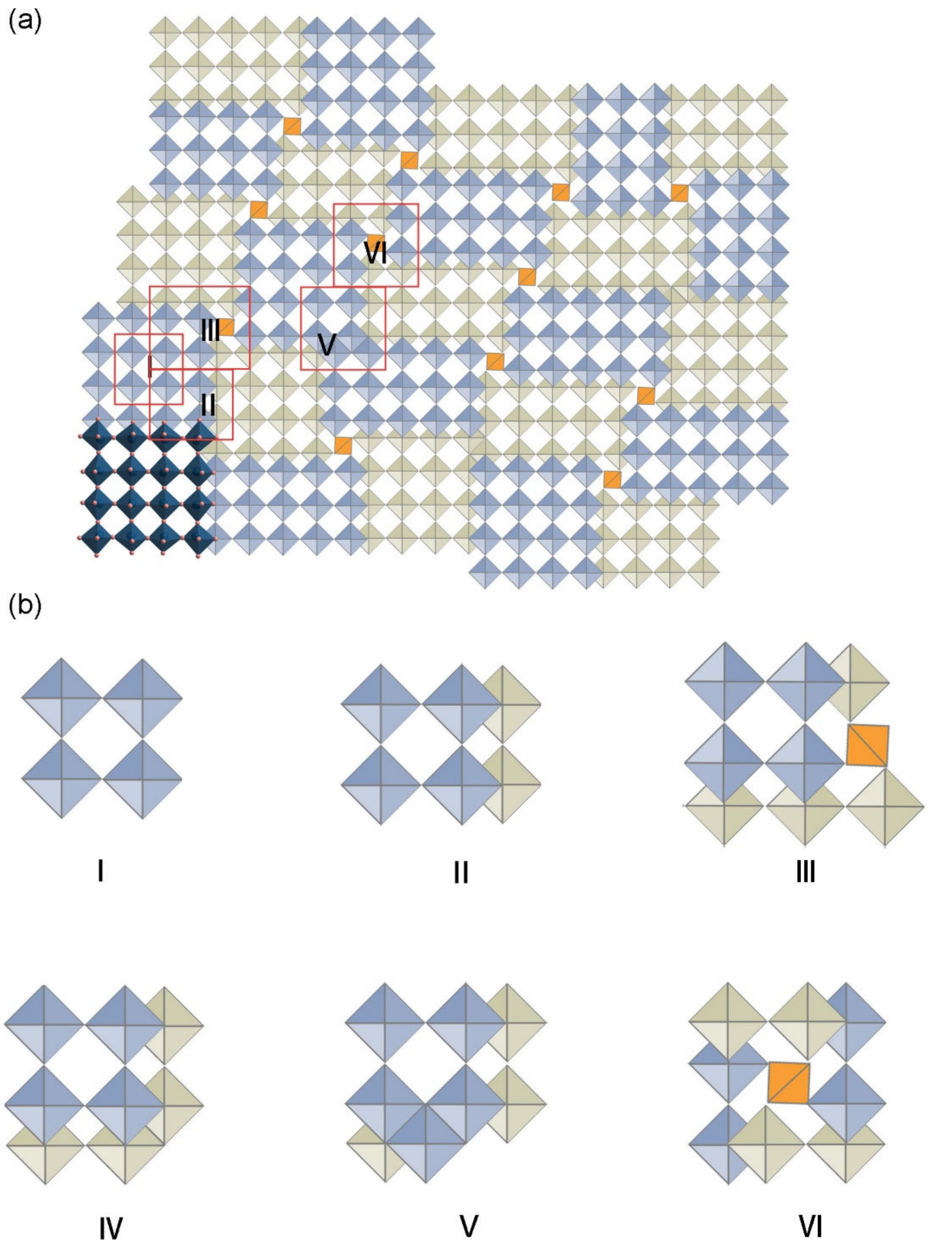

Supplementary Figure 21. (a) Structural illustration of *dt*-$Nb_{12}WO_{33}$ along *a* axis and *b* axis. (b) The six cavity types for Li-ions in Wadsley-Roth crystallographic shear structures; blue and green squares represent the octahedral sites, while orange squares denote tetrahedra sites. (I) Basic ReO_3 type cavity. (II) Singly-capped ReO_3 type cavity. (III-V) Variations of doubly capped cavities. (VI) Tetrahedral site cavity[2].

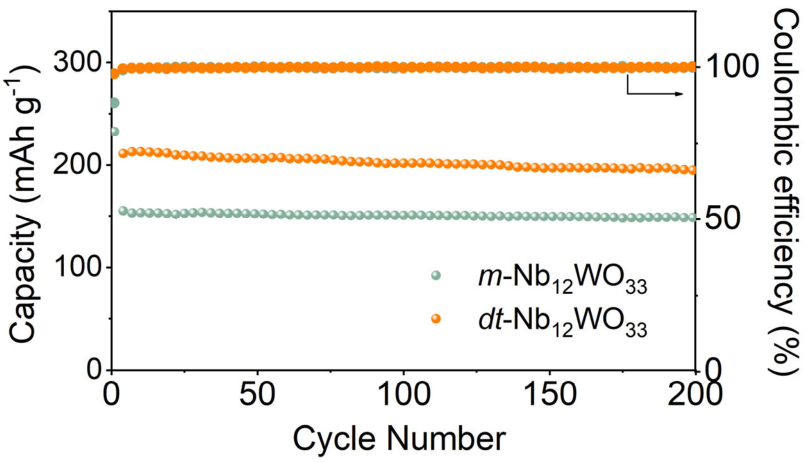

Supplementary Figure 22. Long-term cycling performance of *m*-$Nb_{12}WO_{33}$ and *dt*-$Nb_{12}WO_{33}$ at 5 C.

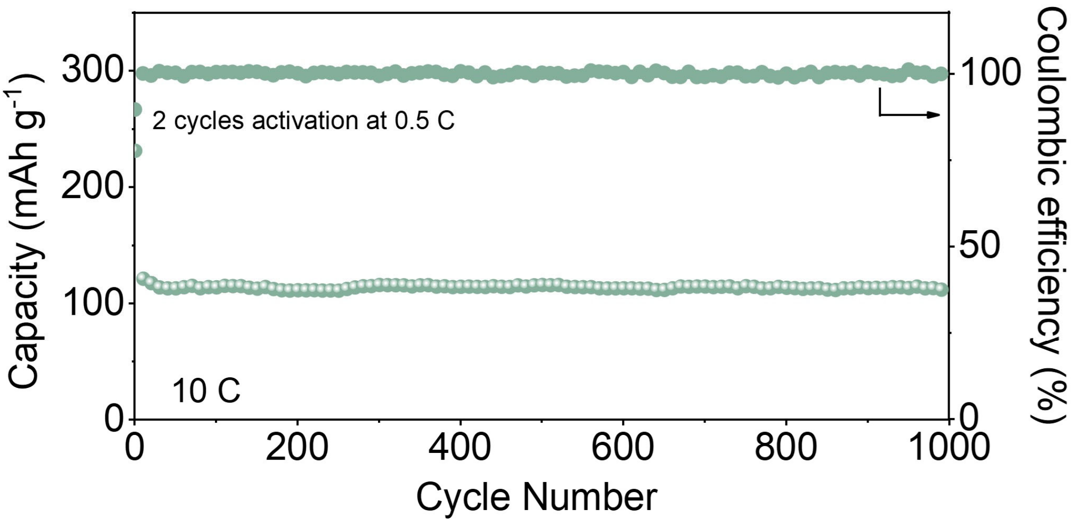

Supplementary Figure 23. Long-term cycling performance of *m*-$Nb_{12}WO_{33}$ at 10 C.

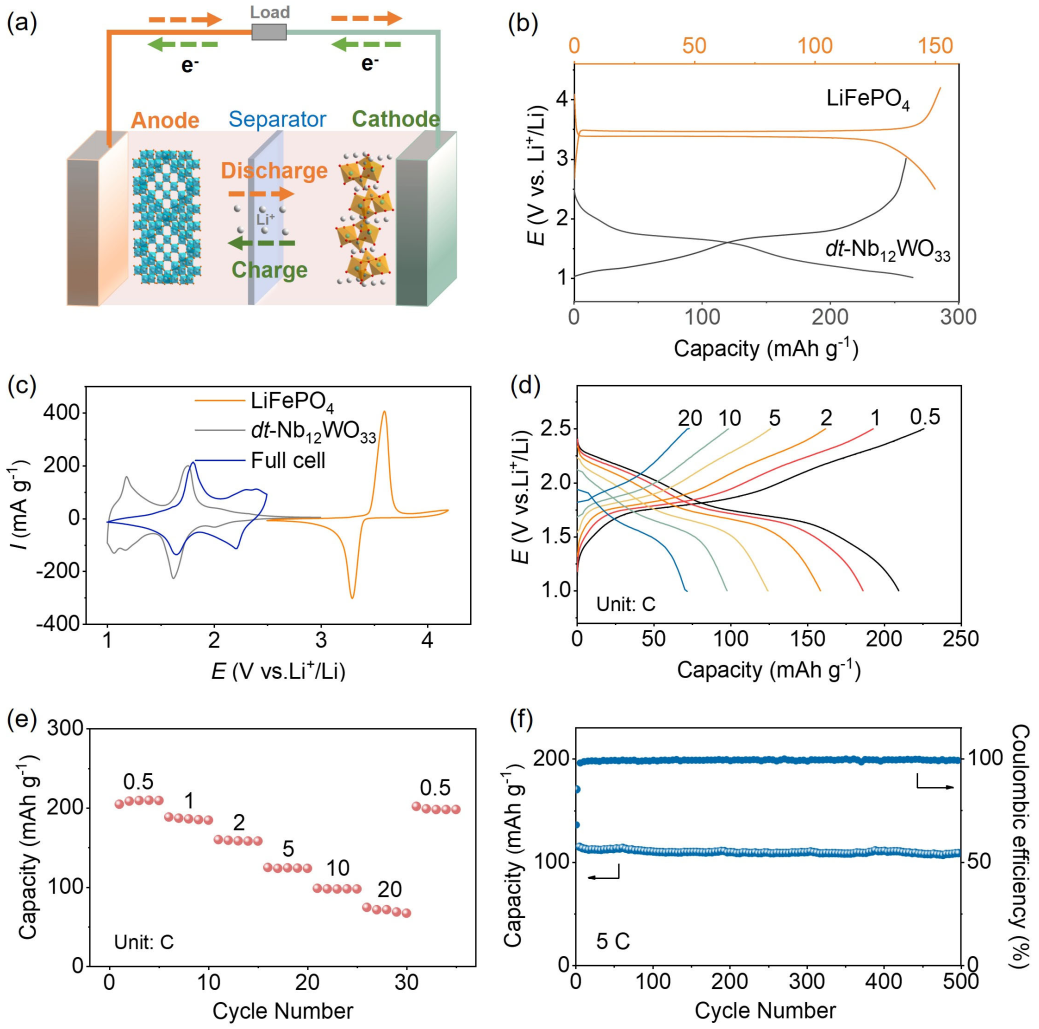

Supplementary Figure 24. Electrochemical performance of *dt*-$Nb_{12}WO_{33}$||LFP full cell. (a) Schematic illustration of the full cell with *dt*-$Nb_{12}WO_{33}$ as negative electrode and commercial LFP as positive electrode. (b) Galvanostatic charge/discharge curves of the *dt*-$Nb_{12}WO_{33}$ and LFP at 0.5 C. (c) CV curves of *dt*-$Nb_{12}WO_{33}$, LFP and *dt*-$Nb_{12}WO_{33}$||LFP full cell at 0.1 mV s^{-1}. (d) Galvanostatic charge/discharge and (e) rate performance of the *dt*-$Nb_{12}WO_{33}$||LFP full cell at various current rates between 1.0 – 2.5 V. (f) Long-term cycling performance of *dt*-$Nb_{12}WO_{33}$||LFP full cell.

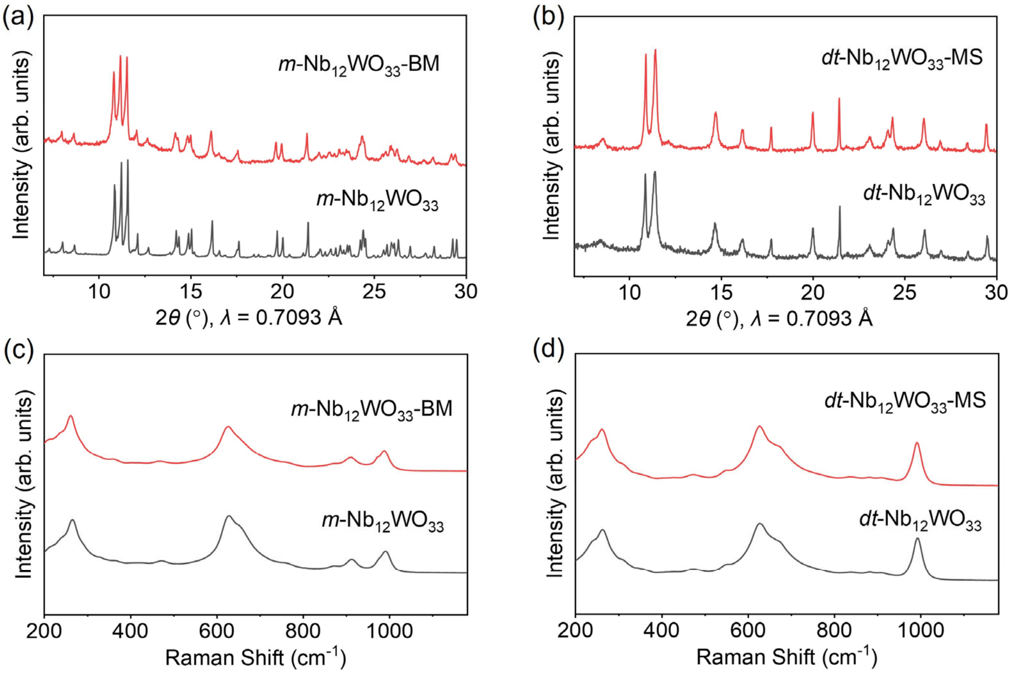

Supplementary Figure 25. XRD patterns of (a) *m*-$Nb_{12}WO_{33}$-BM and *m*-$Nb_{12}WO_{33}$, and (b) *dt*-$Nb_{12}WO_{33}$-MS and *dt*-$Nb_{12}WO_{33}$. Raman spectra of (c) *m*-$Nb_{12}WO_{33}$-BM and *m*-$Nb_{12}WO_{33}$, and (d) *dt*-$Nb_{12}WO_{33}$-MS and *dt*-$Nb_{12}WO_{33}$.

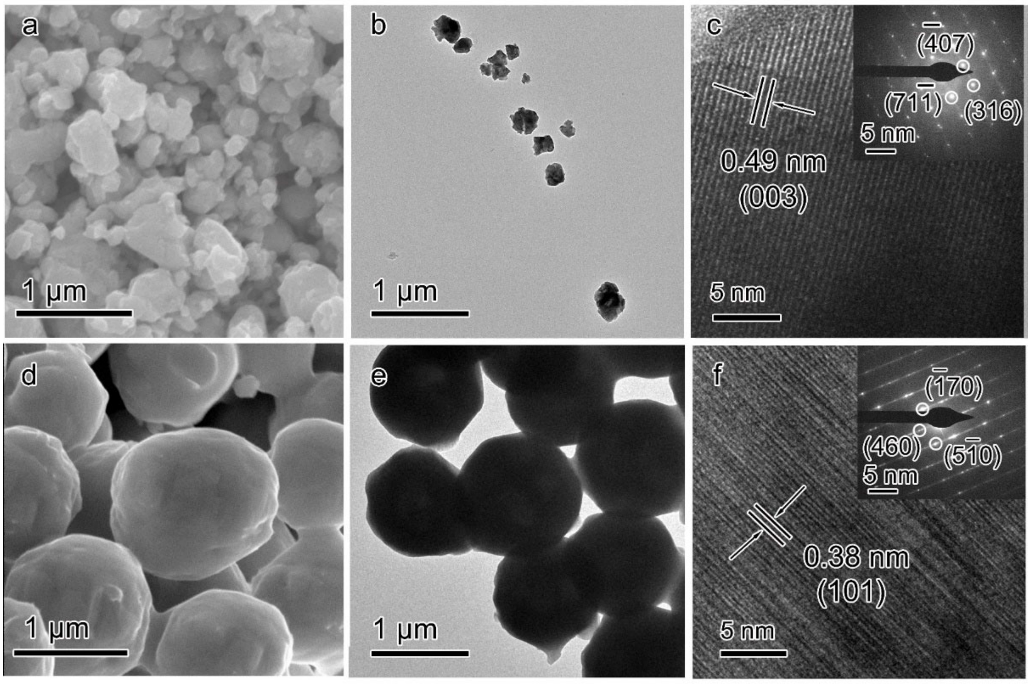

Supplementary Figure 26. (a) SEM, (b) TEM, (c) HR-TEM and SAED images of *m*-$Nb_{12}WO_{33}$-BM. (d) SEM, (e) TEM, (f) HR-TEM and SAED images of *dt*-$Nb_{12}WO_{33}$-MS.

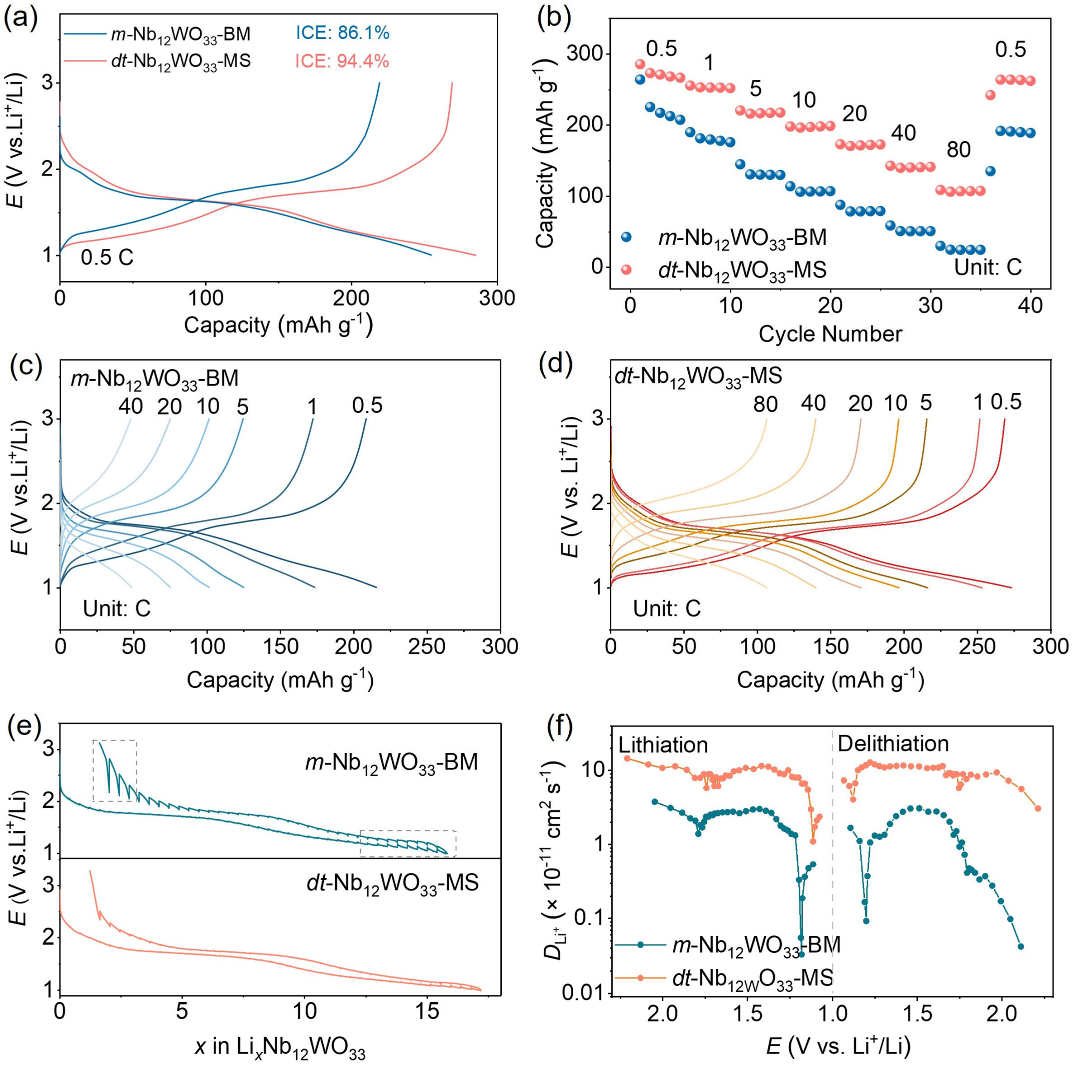

Supplementary Figure 27. (a) Initial discharge-charge profiles of *m*-$Nb_{12}WO_{33}$-BM and *dt*-$Nb_{12}WO_{33}$-MS. (b) Rate capability of *m*-$Nb_{12}WO_{33}$-BM and *dt*-$Nb_{12}WO_{33}$-MS. Discharge-charge profiles of (c) *m*-$Nb_{12}WO_{33}$-BM and (d) *dt*-$Nb_{12}WO_{33}$-MS at various rates. (e) GITT profiles of *m*-$Nb_{12}WO_{33}$-BM and *dt*-$Nb_{12}WO_{33}$-MS as a function of x in $Li_xNb_{12}WO_{33}$. (f) Variation of the D_{Li+} determined by GITT with potential.

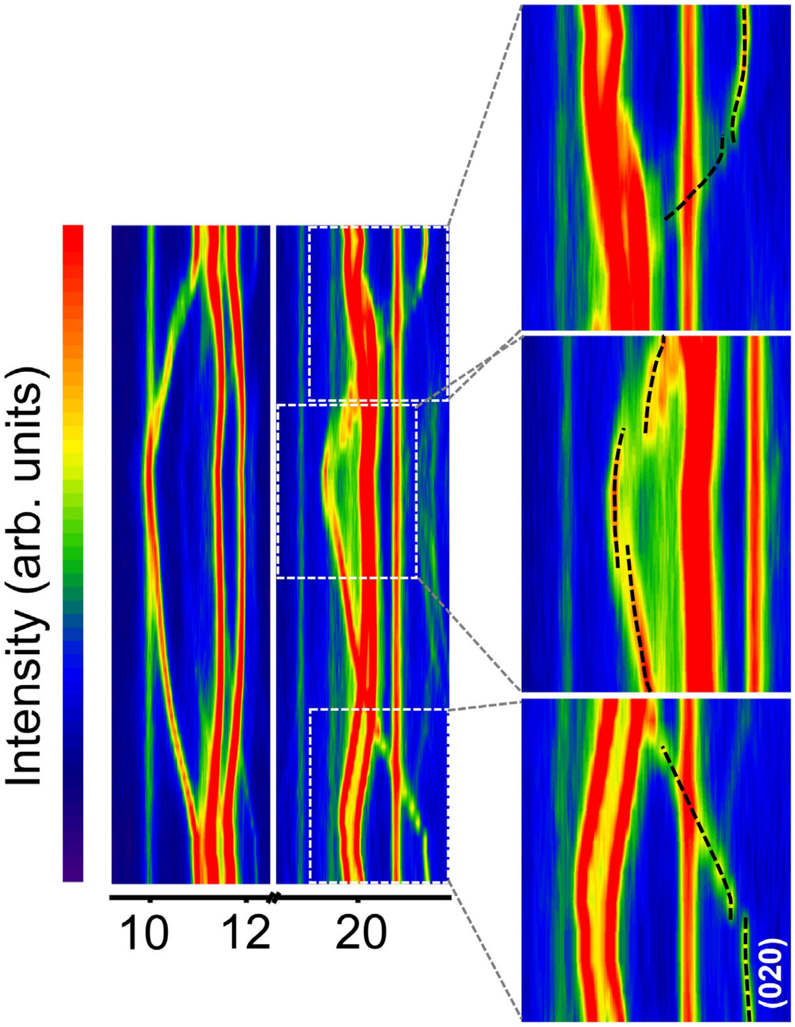

Supplementary Figure 28. *Operando* XRD contour plots of *m*-$Nb_{12}WO_{33}$ at selected angle ranges.

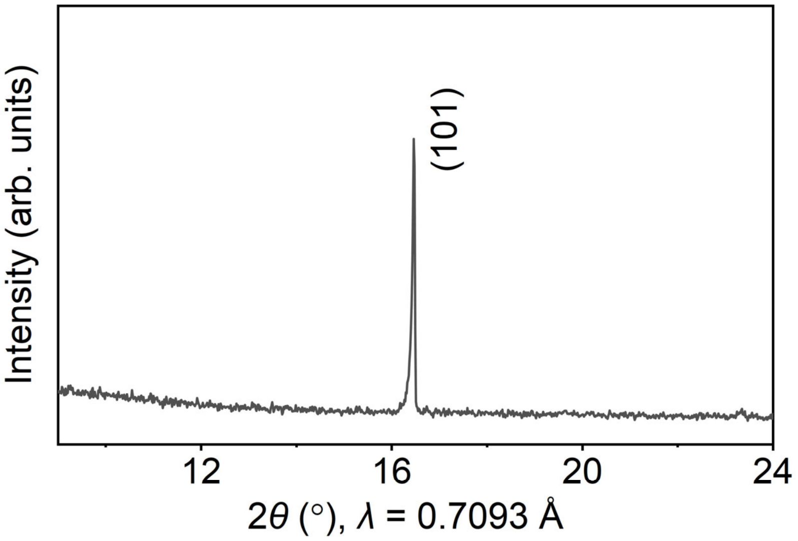

Supplementary Figure 29. XRD pattern of lithium metal.

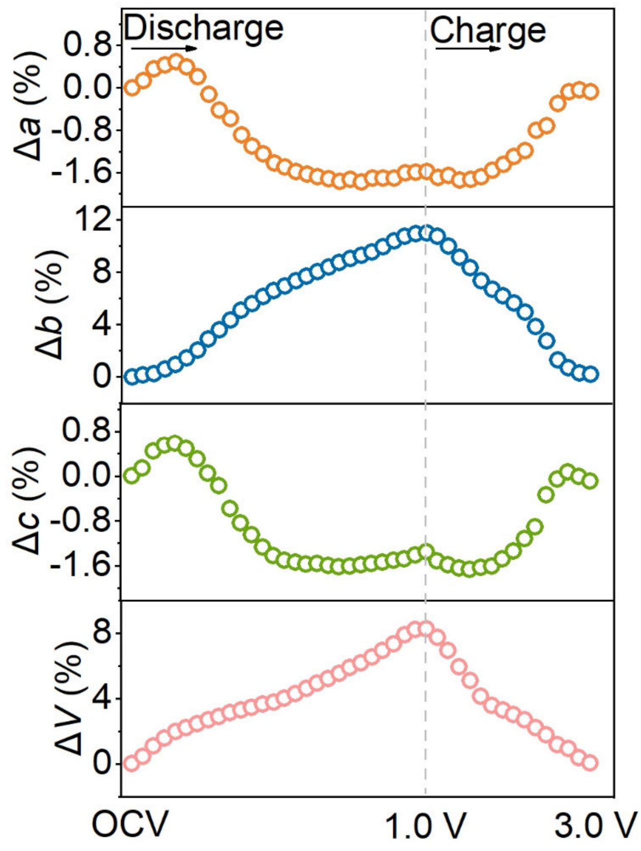

Supplementary Figure 30. Evolution of the unit cell parameters during Li^+ insertion and extraction for the *m*-$Nb_{12}WO_{33}$ electrode.

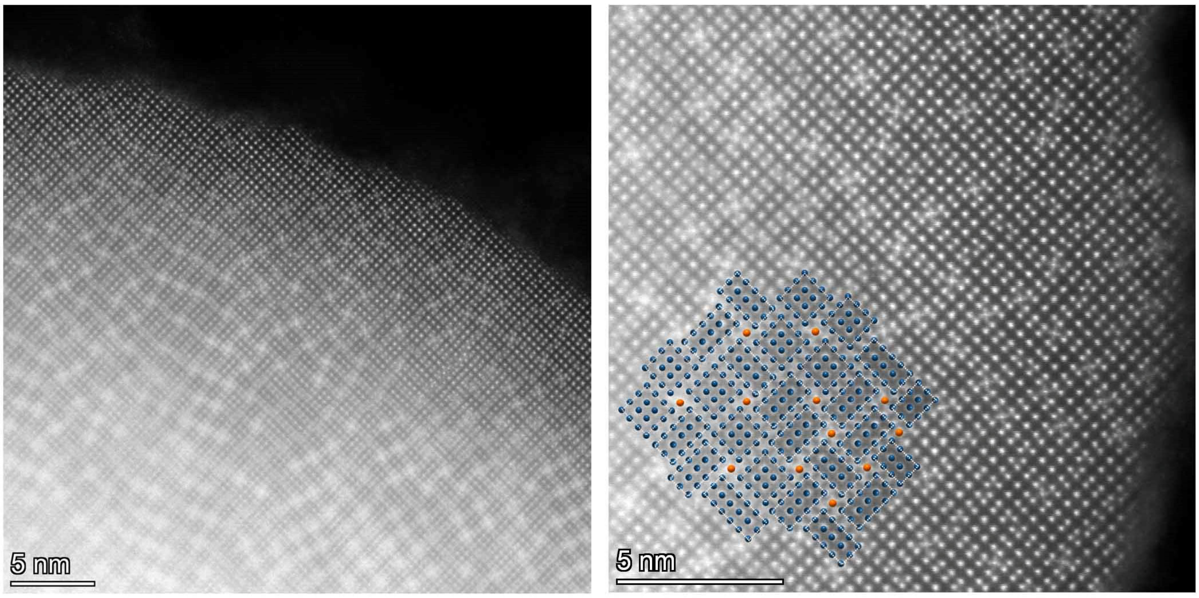

Supplementary Figure 31. AC-STEM images of lithiated (discharged to 1.0 V) *dt*-$Nb_{12}WO_{33}$ viewed along the [001] zone axis after the second cycle (metals in octahedral sites and tetrahedral sites are indicated by blue and orange spheres, respectively).

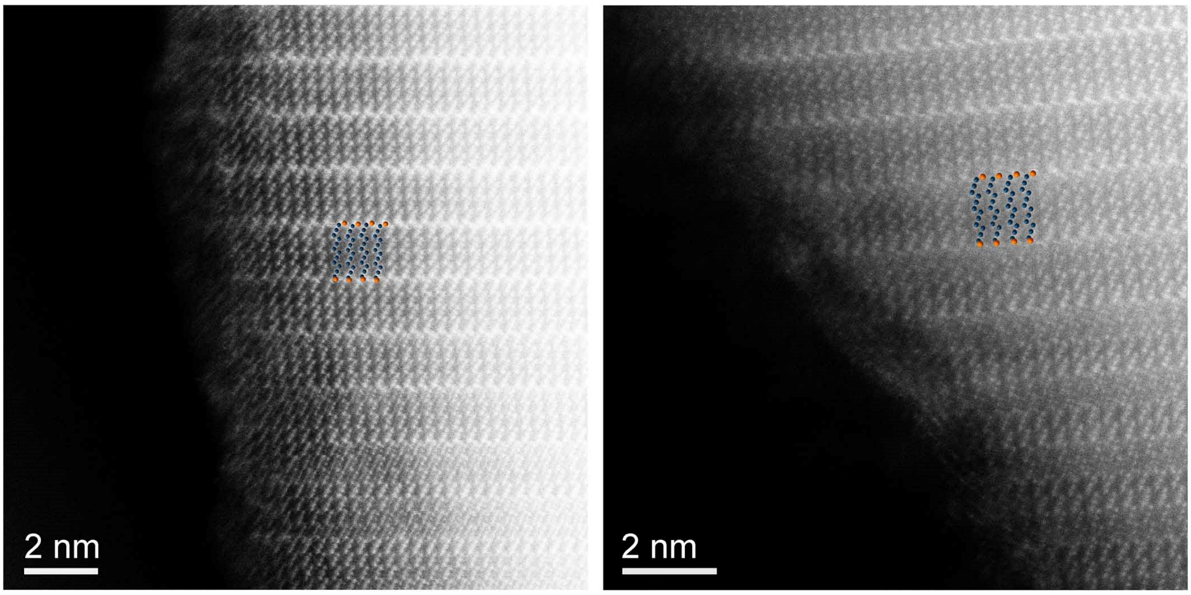

Supplementary Figure 32. AC-STEM images of lithiated *m*-$Nb_{12}WO_{33}$ viewed along the [1-10] zone axis after the second cycle (metals in octahedral sites and tetrahedral sites are indicated by blue and orange spheres, respectively).

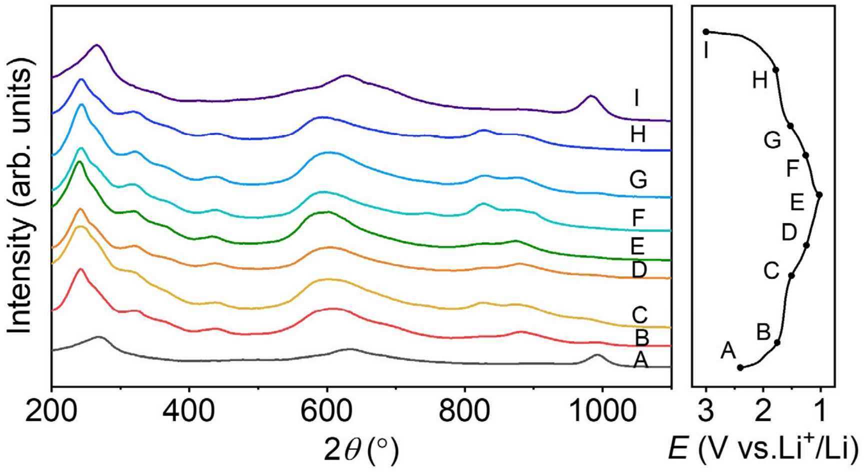

Supplementary Figure 33. *Ex situ* Raman spectra of *dt*-$Nb_{12}WO_{33}$ at different states during the first discharge and charge processes.

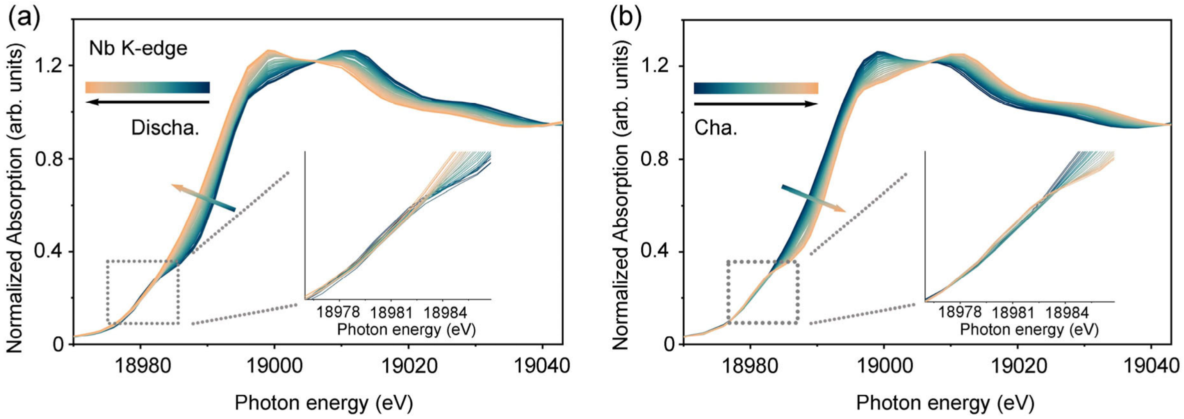

Supplementary Figure 34. *Operando* Nb K-edge XANES spectra of *m*-$Nb_{12}WO_{33}$ for (a) the discharge and (b) charge processes.

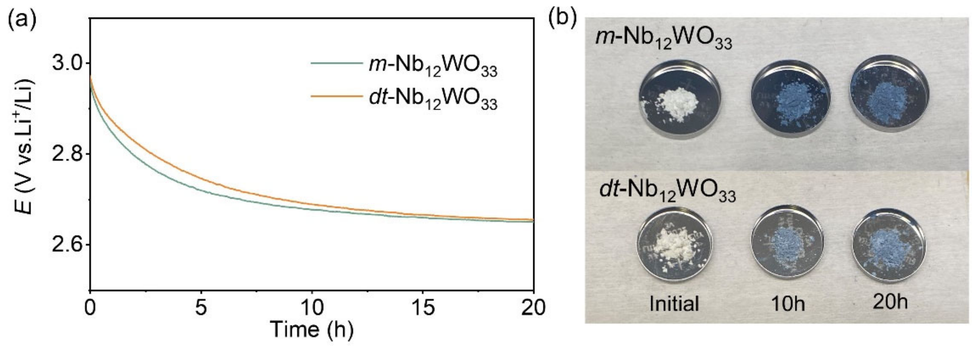

Supplementary Figure 35. (a) Voltage profiles of *m*-$Nb_{12}WO_{33}$ and *dt*-$Nb_{12}WO_{33}$ as a function of time. (b) Images of *m*-$Nb_{12}WO_{33}$ and *dt*-$Nb_{12}WO_{33}$ showing the change in the color of the materials from the initial white to light blue with time.

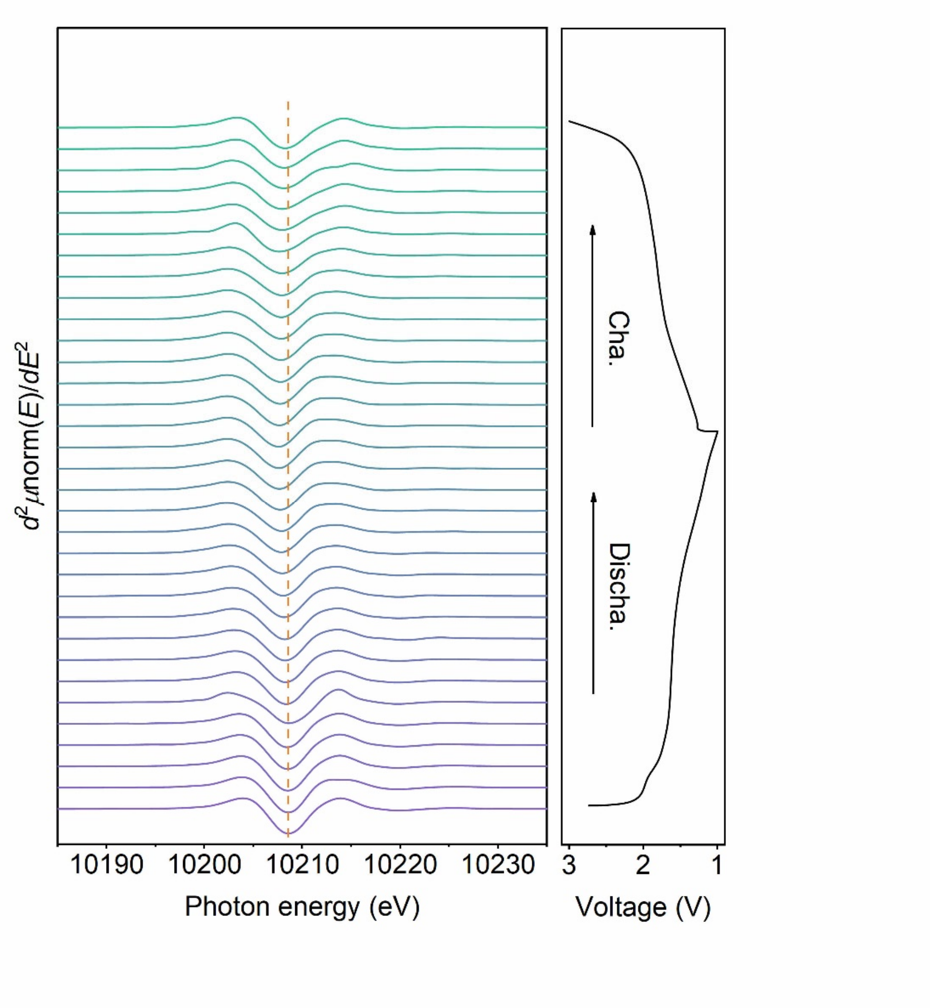

Supplementary Figure 36. Second derivatives of the *operando* W L_{III}-edge XANES spectra of *m*-$Nb_{12}WO_{33}$ upon Li^+ insertion and extraction.

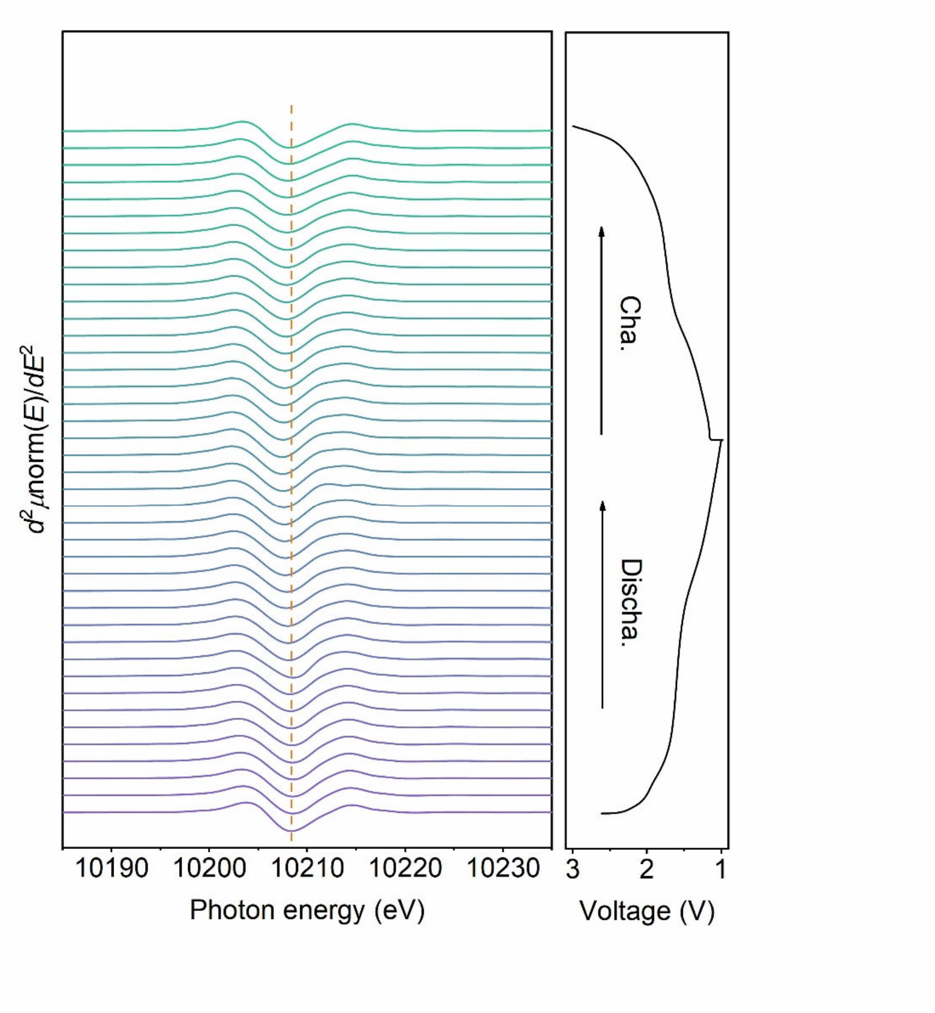

Supplementary Figure 37. Second derivatives of the *operando* W L_{III}-edge XANES spectra of *dt*-$Nb_{12}WO_{33}$ upon Li^+ insertion and extraction.

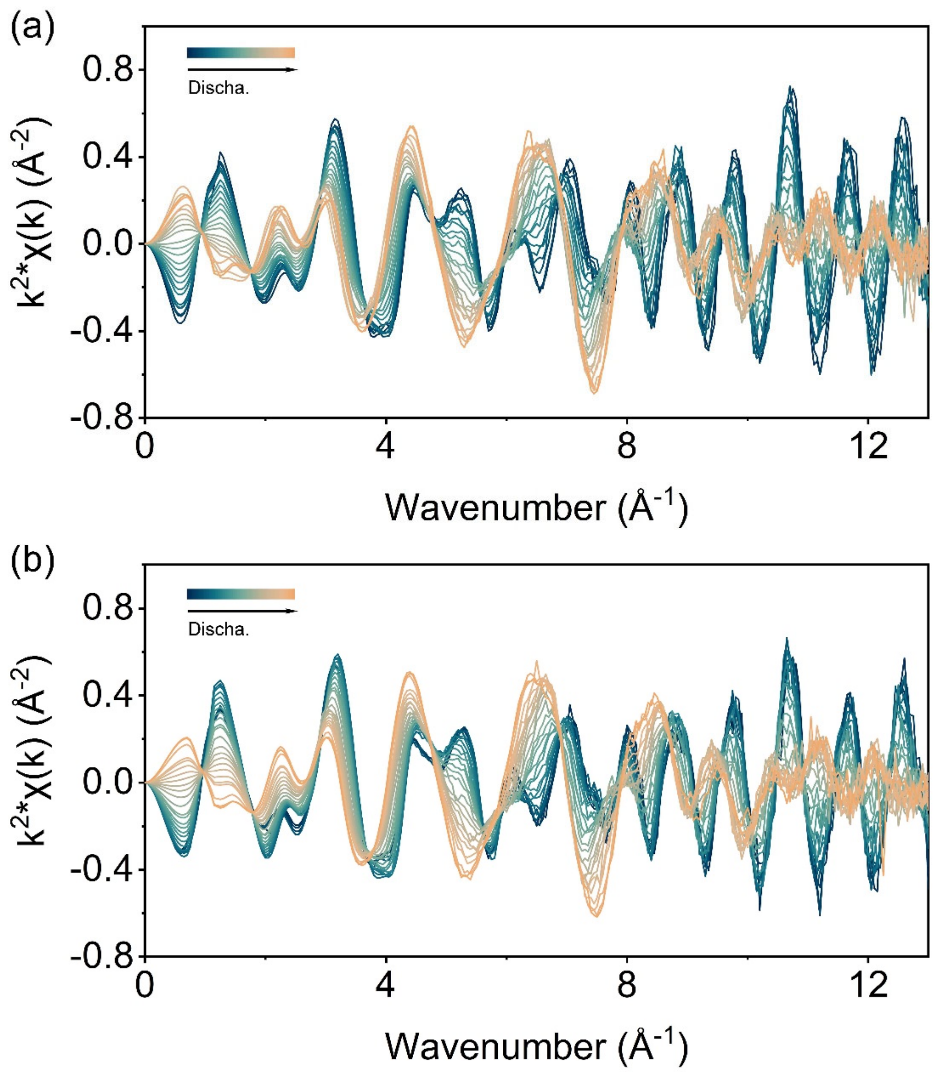

Supplementary Figure 38. *Operando* κ^2-weighted Nb K-edge EXAFS oscillations of (a) *m*-$Nb_{12}WO_{33}$ and (b) *dt*-$Nb_{12}WO_{33}$.

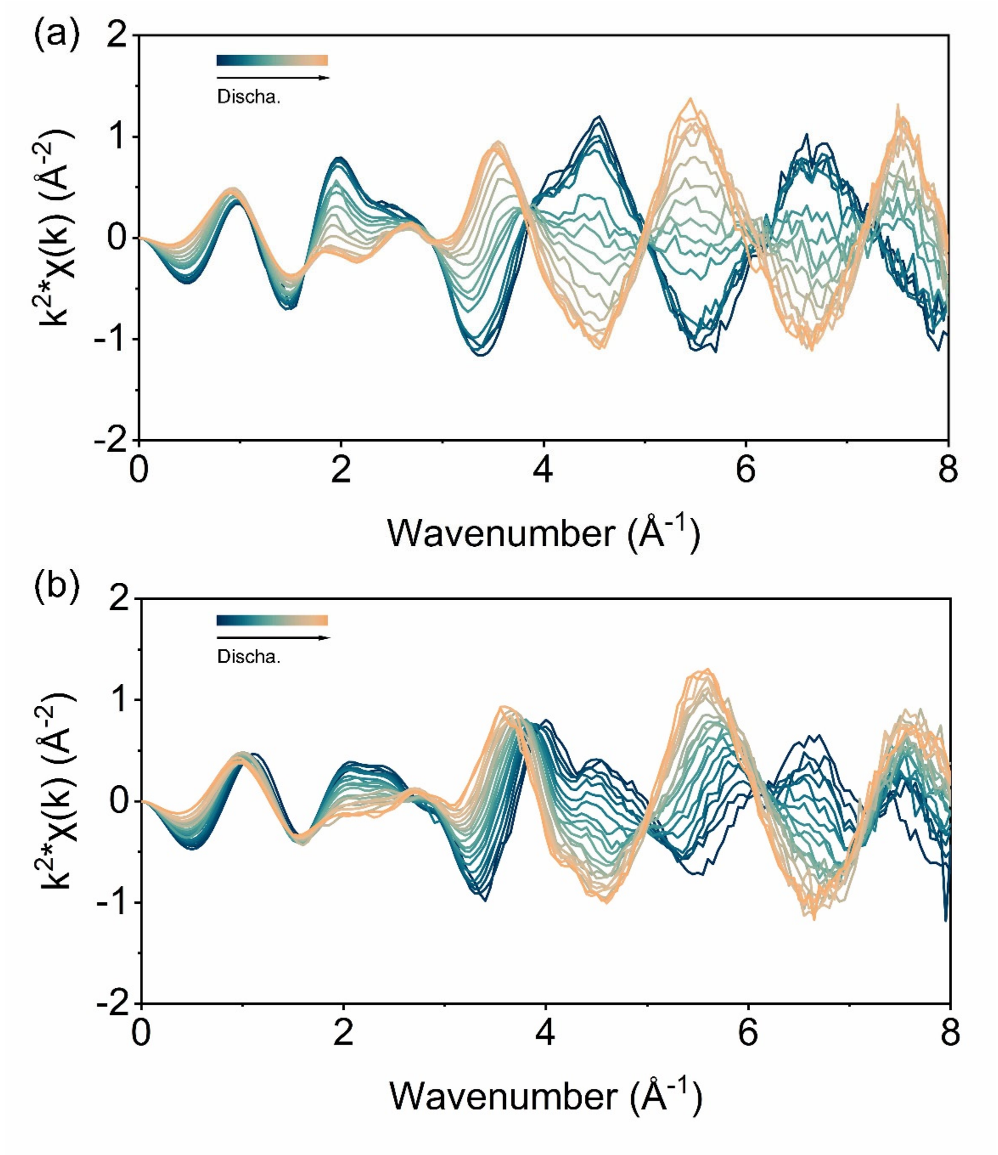

Supplementary Figure 39. *Operando* κ^2-weighted W L_{III}-edge EXAFS oscillations of (a) *m*-$Nb_{12}WO_{33}$ and (b) *dt*-$Nb_{12}WO_{33}$.

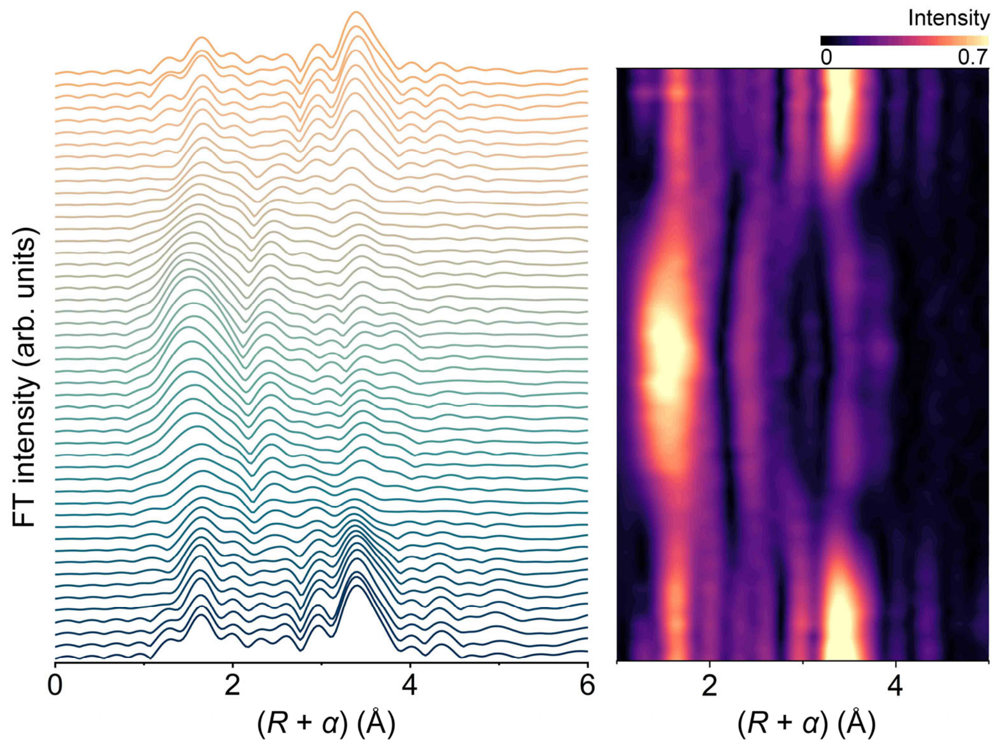

Supplementary Figure 40. *Operando* Nb K-edge EXAFS spectra and contour plot of *m*-$Nb_{12}WO_{33}$.

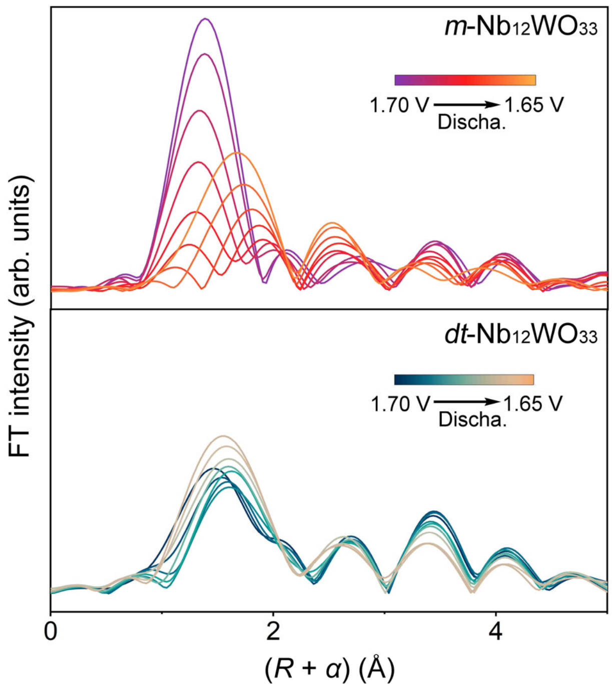

Supplementary Figure 41. *Operando* W L_{III}-edge EXAFS spectra during the two-phase transition reaction of *m*-$Nb_{12}WO_{33}$ and *dt*-$Nb_{12}WO_{33}$within the voltage range 1.65-1.70 V.

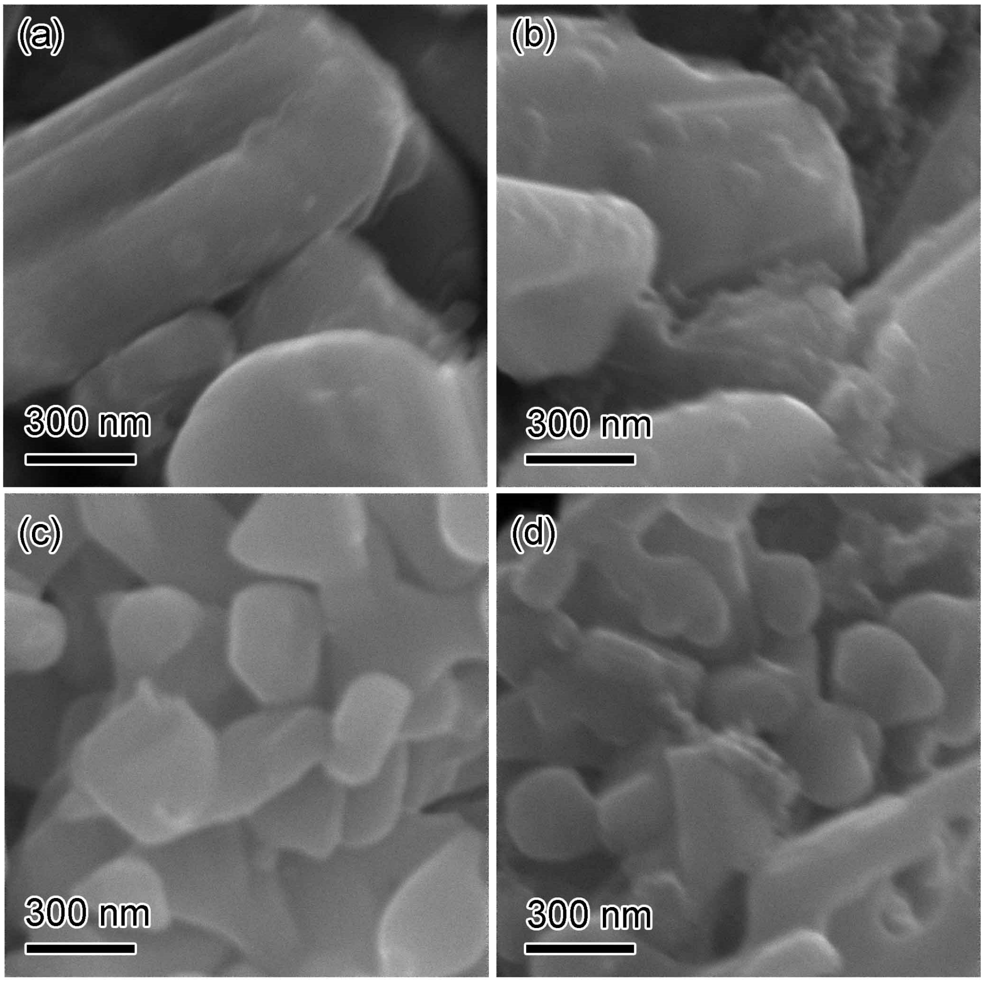

Supplementary Figure 42. SEM images of the (a, b) *m*-$Nb_{12}WO_{33}$ and (c, d) *dt*-$Nb_{12}WO_{33}$ electrode after 500 cycles at 10 C.

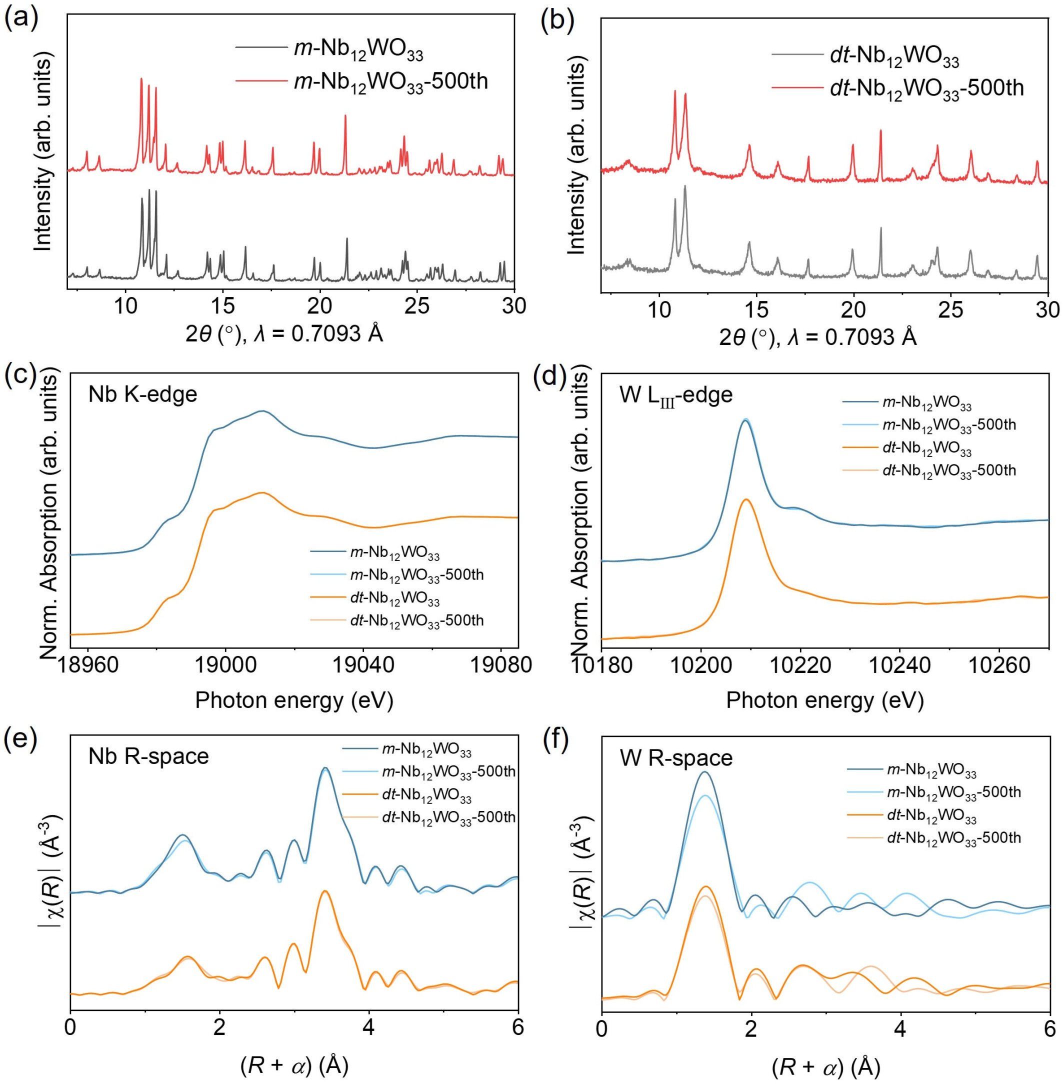

Supplementary Figure 43. XRD patterns of (a) *m*-$Nb_{12}WO_{33}$ and (b) *dt*-$Nb_{12}WO_{33}$ before and after 500 cycles at 10 C. (c) Nb K-edge and (d) W L_{III}-edge XANES spectra of *m*-$Nb_{12}WO_{33}$ and *dt*-$Nb_{12}WO_{33}$ before and after 500 cycles at 10 C. (e) Nb K-edge and (f) W L_{III}-edge EXAFS spectra of *m*-$Nb_{12}WO_{33}$ and *dt*-$Nb_{12}WO_{33}$ before and after 500 cycles at 10 C.

Supplementary Table 1. Crystallographic parameters for *m*-$Nb_{12}WO_{33}$.

chemical formula	$Nb_{1.50}W_{0.12}O_{4.12}$
crystal system	monoclinic
space group	*C* 2/m
a (Å)	22.3002(15)
b (Å)	3.8279(2)
c (Å)	17.7490(12)
α (deg)	90
β (deg)	123.338(2)
V (Å)	1265.78(14)
Z	16
density (calculated) (g/cm^3)	4.793
Temp. (K)	101(2)
abs. coff. (mm^{-1})	9.865
F(0 0 0)	1660
Theta range for data collection (deg)	2.186 to 27.521
reflections collected	14015
Independent reflections	1664 [R(int) = 0.0825]
Completeness to theta = 25.242°	99.6%
refinement method	full-matrix least-squares on F^2
data / restraints / parameters	1664 / 0 / 92
goodness-of-fit on F2	1.038
final R indices [I>2sigma(I)]	*R*1 = 0.0383, w*R*2 = 0.0851
R indices (all data)	*R*1 = 0.0484, w*R*2 = 0.0932
largest diff. peak and hole ($Å^{-3}$)	1.820/-1.590

	x	*y*	*z*	occ.	U_{iso}
Nb1	0.41697(4)	0	0.38056(5)	1	0.0145(2)
Nb2	0.46442(4)	0	0.71313(5)	1	0.00531(16)

Nb3	0.29725(4)	0	0.47235(5)	1	0.00553(16)
Nb4	0.24819(4)	0	0.13672(5)	1	0.00485(16)
Nb5	0.12922(4)	0	0.23061(5)	1	0.00460(16)
Nb6	0.63319(4)	0	0.95611(5)	1	0.00455(16)
W1	0	-0.2500(2)	0	0.4438(16)	0.0049(2)
O1	0.6975(3)	0	0.9200(4)	1	0.0047(12)
O2	0.5250(3)	0	0.6749(4)	1	0.0071(12)
O3	0.3508(3)	0	0.4238(4)	1	0.0098(13)
O4	0.1785(3)	0	0.1766(4)	1	0.0068(12)
O5	0.5496(3)	0	0.8452(4)	1	0.0036(11)
O6	0.3777(3)	0	0.5959(4)	1	0.0071(12)
O7	0.2052(3)	0	0.3488(4)	1	0.0099(13)
O8	0.3250(3)	0	0.2493(4)	1	0.0098(13)
O9	0.5	0	0.5	1	0.0126(19)
O10	0.2779(3)	-0.5	0.4805(4)	1	0.0053(12)
O11	0.4515(3)	-0.5	0.7301(4)	1	0.0035(11)
O12	0.0320(3)	0	0.0991(4)	1	0.0067(12)
O13	0.0733(3)	-0.5	0.0168(4)	1	0.0053(12)
O14	0.1039(3)	-0.5	0.2317(4)	1	0.0073(12)
O15	0.2249(3)	-0.5	0.1039(4)	1	0.0065(12)
O16	0.6496(3)	-0.5	0.9922(4)	1	0.0077(12)
O17	0.4105(3)	-0.5	0.3705(4)	1	0.0123(14)

Supplementary Table 2. Amounts of Nb and W in *m*-$Nb_{12}WO_{33}$ and *dt*-$Nb_{12}WO_{33}$ determined by ICP-OES.

	Nb	W
m-$Nb_{12}WO_{33}$	12.06	0.94
dt-$Nb_{12}WO_{33}$	12.14	0.86

Supplementary Table 3. Curve fitting parameters of the W L_{III}-edge EXAFS spectrum of *m*-$N_{12}WO_{33}$.

Path	d / Å	N	R / Å	σ^2 / Å
W-O	1.70	4	1.76(1)	0.001(1)

S_0^2 for this fit is 0.8. ΔE_0 was refined as a global fit parameter. Data ranges: $2 \leq k \leq 12$ Å^{-1}, $1.1 \leq R \leq 2.3$ Å. The number of variable parameters is 4, out of a total of independent data points of 8.6. R factor for the fitting is 0.8 %.

Supplementary Table 4. Curve fitting parameters of the W L_{III}-edge EXAFS spectrum of *dt*-$N_{12}WO_{33}$.

Path	d / Å	N	R / Å	σ^2 / Å
W-O	1.70	2.6	1.77(1)	0.001(1)

S_0^2 for this fit is 0.8. ΔE_0 was refined as a global fit parameter. Data ranges: $2 \leq k \leq 12$ Å^{-1}, $1.1 \leq R \leq 2.3$ Å. The number of variable parameters is 4, out of a total of 8.6 independent data points. R factor for the fitting is 1.2 %.

Supplementary Table 5. Curve fitting parameters of the Nb K-edge EXAFS spectrum of *m*-$Nb_{12}WO_{33}$.

Path	*d* / Å	*N*	*R* / Å	σ^2 / Å
Nb-O1	1.80	1.5	1.82(1)	0.004(2)
Nb-O2	1.95	2	1.97(1)	0.004(2)
Nb-O3	1.98	2	2.00(1)	0.004(2)
Nb-Nb1	3.36	2	3.37(1)	0.004
Nb-W1	3.60	1	3.61(1)	0.004
Nb-Nb2	3.83	3	3.84(1)	0.004
Nb-O-Nb1	3.85	4	3.84(1)	0.008(1)
Nb-O-Nb2	3.88	4	3.86(1)	0.008(1)
Nb-O-Nb-O	3.85	2	3.84(1)	0.008(1)
Nb-O3	3.92	4	3.87(4)	0.013(8)
O-Nb-O	4.31	4	4.26(4)	0.013(8)
Nb-W2	4.82	1	4.81(16)	0.012(16)

S_0^2 of the first shell for this fit is 0.5. ΔE_0 was refined as a global fit parameter. Data ranges: $2 \leqslant k \leqslant 14$ Å^{-1}, $1.52 \leqslant R \leqslant 5.2$ Å. The number of variable parameters is 12, out of a total of 28.1 independent data points. R factor for the fitting is 2.0 %. The Debye-Waller factors were constrained as follows to reduce the number variables: σ^2 (Nb-O1) = σ^2 (Nb-O2) = σ^2 (Nb-O3), σ^2(Nb-Nb1) = σ^2(Nb-W1) = σ^2(Nb-Nb2), σ^2(Nb-O3) = σ^2(O-Nb-O), σ^2(Nb-O-Nb1) = σ^2(Nb-O-Nb2) = σ^2(Nb-O-Nb-O).

Supplementary Table 6. Curve fitting parameters of the Nb K-edge EXAFS spectrum of *dt*-$N_{12}WO_{33}$.

Path	*d* / Å	*N*	*R* / Å	σ^2 / Å
Nb-O1	1.80	1.5	1.83(1)	0.004(2)
Nb-O2	1.97	3	2.00(1)	0.004(2)
Nb-Nb1	3.36	4	3.37(1)	0.006(1)
Nb-W1	3.60	1	3.61(1)	0.006(1)
Nb-Nb2	3.83	4	3.85(1)	0.006(1)
Nb-O-Nb1	3.84	4	3.81(1)	0.004
Nb-O-Nb2	3.89	4	3.85(1)	0.004
Nb-O-Nb-O	3.85	2	3.82(1)	0.004
Nb-O3	3.92	4	3.91(6)	0.013(14)
Nb-O4	4.09	4	4.07(6)	0.013(14)
O-Nb-O	4.31	4	4.29(6)	0.013(14)
Nb-W2	4.50	1	4.48(7)	0.009(7)
Nb-W3	4.82	1	4.80(7)	0.009(7)

S_0^2 of the first shell for this fit is 0.5. ΔE_0 was refined as a global fit parameter. Data ranges: $2 \leqslant k \leqslant 14$ Å, $1.52 \leqslant R \leqslant 5.2$ Å. The number of variable parameters is 12, out of a total of 28.1 independent data points. R factor for the fitting is 2.0 %. The debye-Waller factors were constrained as follows to reduce the number variables: σ^2 (Nb-O1) = σ^2 (Nb-O2), σ^2(Nb-Nb1) = σ^2(Nb-W1) = σ^2(Nb-Nb2), σ^2(Nb-O3) = σ^2(Nb-O4) = σ^2(O-Nb-O), σ^2(Nb-O-Nb1) = σ^2(Nb-O-Nb2) = σ^2(Nb-O-Nb-O) and σ^2(Nb-W1) = σ^2(Nb-W2).

Supplementary Table 7. Comparison of the electrochemical performance of *dt*-$N_{12}WO_{33}$ with that of other previously reported Nb-based materials.

Material	Structure type	Voltage range (V vs. Li^+/Li)	Initial reversible capacity (mAh g^{-1})	Rate performance (mAh g^{-1})	Current density of rate performance (A g^{-1})	Carbon content (%)	Mass loading (mg cm^{-2})
$Nb_{16}W_5O_{55}$[3]	Shear structure	1.0-3.0	225 (at 34.3 mA g^{-1})	~50	10.3	10	2-3
$Nb_{18}W_{16}O_{93}$[3]	Tungsten bronze structure	1.0-3.0	205 (at 29.8 mA g^{-1})	70	14.9	10	2-3
$Nb_{14}W_3O_{44}$[4]	Shear structure	1.0-3.0	221.3 (at 89 mA g^{-1})	84.4	8.9	20	1.4-1.7
Nano-block $Nb_{14}W_3O_{44}$[5]	Shear structure	1.0-3.0	241.1 (at 89 mA g^{-1})	109.5	14.2	10	2
$Nb_{12}WO_{33}$ nanowires[6]	Shear structure	1.0-3.0	228 (at 200 mA g^{-1})	145.8	0.7	10	1.5
$Nb_{18}W_8O_{69}$[7]	Shear structure	1.0-3.0	~230 (at 32.8 mA g^{-1})	~30	9.8	10	2.0 ± 0.2
H-Nb_2O_5[8]	Shear structure	1.0-3.0	269 (at 20 mA g^{-1})	94	4	20	1.0-1.4
PNb_9O_{25}[9]	Shear structure	1.0-3.0	~235 (at 25.4 mA g^{-1})	30	15.2	15	1.5
VNb_9O_{25}[9]	Shear structure	1.0-3.0	~180 (at 29.2 mA g^{-1})	25	11.7	15	1.5
$TiNb_{24}O_{62}$[10]	Shear structure	1.0-3.0	214 (at 20.5 mA g^{-1})	~100	3.1	10	2-3
$V_7Nb_6O_{29}$[11]	Shear structure	1.2-3.3	208 (at 20 mA g^{-1})	106	2	10	1-1.5
$Ba_{3.4}Nb_{10}O_{28.4}$[12]	Tungsten bronze structure	0.8-3.0	167 (at 28.97 mA g^{-1})	82	2.9	20	\

$Nb_{12}W_{11}O_{63}$[13]	Tungsten bronze structure	1.3-3	176 (at 22.6 mA g^{-1})	100	4.5	10	2
$CoNb_{11}O_{29}$[14]	Shear structure	0.8-3	265 (at 40 mA g^{-1})	113	4	20	1.3
$Nb_{12}O_{29}$[15]	Shear structure	1.0-2.5	243 (at 6.6 mA g^{-1})	60	0.132	20	10-20
dt-$Nb_{12}WO_{33}$ (This work)	Shear structure	1-3	257 (at 95.4 mA g^{-1})	119.5	15.3	20	1.5-2

Supplementary References

1. Weppner, W., & Huggins, R. A. Determination of the kinetic parameters of mixed-conducting electrodes and application to the system Li_3Sb. *J. Electrochem. Soc.*, **124**, 1569 (1977).
2. Cava, R. J., Murphy, D. W., & Zahurak, S. M. Lithium insertion in Wadsley-Roth phases based on niobium oxide. *J. Electrochem. Soc.* **130**, 2345, (1983).
3. Griffith, K. J., Wiaderek, K. M., Cibin, G., Marbella, L. E. & Grey, C. P. Niobium tungsten oxides for high-rate lithium-ion energy storage. *Nature* **559**, 556-563 (2018).
4. Yang, Y. et al. Achieving Achieving ultrahigh-rate and high-safety Li^+ storage based on interconnected tunnel structure in micro-size niobium tungsten oxides. *Adv. Mater.* **32**, 1905295 (2020).
5. Guo, C. et al. Nano-sized niobium tungsten oxide anode for advanced fast-charge lithium-ion batteries. *Small* **18**, 2107365 (2022).
6. Yan, L. et al. Electrospun $WNb_{12}O_{33}$ nanowires: superior lithium storage capability and their working mechanism. *J. Mater. Chem. A* **5**, 8972-8980 (2017).
7. Griffith, K. J., & Grey, C. P. Superionic lithium intercalation through 2× 2 nm^2 columns in the crystallographic shear phase $Nb_{18}W_8O_{69}$. *Chem. Mater.* **32**, 3860-3868 (2020).
8. Yang, M., Li, S., Huang J. Three-dimensional cross-linked Nb_2O_5 polymorphs derived from cellulose substances: Insights into the mechanisms of Lithium storage. *ACS Appl. Mater. Interfaces* **13**, 39501−39512 (2021).

9. Preefer, M. B. et al. Multielectron redox and insulator-to-metal transition upon lithium insertion in the fast-charging, Wadsley-Roth phase PNb_9O_{25}. *Chem. Mater.* **32**, 4553−4563 (2020).
10. Griffith, K. J., Senyshyn A., Grey, C. P. Structural stability from crystallographic shear in TiO_2–Nb_2O_5 phases: cation ordering and lithiation behavior of $TiNb_{24}O_{62}$. *Inorg. Chem.* **56**, 4002−4010 (2017).
11. Lawrence, E. A. et al. Reversible electrochemical lithium cycling in vanadium(IV)- and niobium(V)-based Wadsley–Roth phase. *Chem. Mater.* **35**, 3470−3483 (2023).
12. Xiong, X. et al. Cation-vacancy ordered superstructure enhanced cycling stability in tungsten bronze anode. *Adv. Energy Mater.* **12**, 2201967 (2022).
13. Ma, X.-H., et al. Influence of cut-off voltage on the lithium storage performance of $Nb_{12}W_{11}O_{63}$ anode, *Electrochim. Acta* **332**, 135380 (2020).
14. Liu, H., Chen, C. Wadsley–Roth phase $CoNb_{11}O_{29}$ as a high-performance anode for lithium-ion batteries. *J. Mater. Chem. A*, **12**, 5414-5421 (2024).
15. Li, Y., Sun, C., Goodenough, J. B. Electrochemical Lithium Intercalation in Monoclinic $Nb_{12}O_{29}$. *Chem. Mater.* **23**, 2292–2294 (2011).
